# Effectiveness of Multivitamins vs Folic Acid on Prevention of Neural Tube Defects in Mouse Genetic Models and Human Organoids

**DOI:** 10.1002/advs.202513609

**Published:** 2025-12-08

**Authors:** Huili Li, Jing Zhang, Lori Bulwith, Lee Niswander

**Affiliations:** ^1^ Molecular, Cellular, Developmental Biology Department University of Colorado Boulder 80309 USA

**Keywords:** folic acid, human neural tube organoids, mitosis, multivitamin, neural tube defect

## Abstract

Maternal consumption of folic acid (FA)‐containing multivitamins/minerals (MVM) are recommended to reduce the risk for neural tube defects (NTDs). However, comparison between MVM supplementation and FA alone relative to NTD risk and possible mechanisms remain unclear. The studies demonstrate MVM can decrease NTD incidence in mouse genetic models with FA‐resistant or FA‐detrimental responses. To evaluate cellular and metabolic impacts, human iPSC‐derived neuroectoderm organoids are generated with an elliptical shape resembling the human neural tube and cranial to upper spinal identity. Upon pharmacological or genetic disruption, both MVM or FA alone can normalize abnormal apical F‐actin, lumen size, and premature neuronal differentiation. However, FA alone enhances DNA synthesis and shortens the cell division rate, while MVM maintains these parameters at control levels. High‐performance liquid chromatography (HPLC) analysis of mouse embryos and human organoids indicates FA causes more variation of the nucleotide pool, whereas MVM maintains homeostasis. Thymine is reduced in FA alone and increased in MVM and thymine/thymidine supplementation can ameliorate FA‐induced hyperactivation of cell division. The in vivo cellular and phenotypic data in human organoids and mice show the effectiveness of MVM supplementation, that in some cases surpasses FA alone, in maintaining cellular homeostasis and preventing NTD.

## Graphical abstract

Folic acid (FA) and multivitamins/minerals (MVM) are recommended for the prevention of neural tube defects (NTDs). Using a novel human neural tube‐like organoid system and mouse models, the study compares the effectiveness of two supplements on NTDs and cellular behaviors. This shows that MVM supplementation is effective in normalizing cell behaviors, nucleotide homeostasis, and reducing NTD risk in FA non‐responsive or FA‐detrimental models.

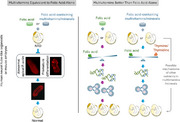

## 1 Introduction

Neural tube defects (NTDs), including anencephaly and spina bifida, are the second‐most common birth defect and can lead to death or life‐long disability. Folic acid (FA) supplementation shows a significant protective effect in clinical trials,^[^ ^1^, ^2^ ^]^ and FA fortification has been implemented by ~80 countries, leading to a substantial improvement in NTD prevalence worldwide.^[^ ^3^, ^4^ ^]^ In the U.S., current recommendations are supplementation with a multivitamin containing 0.4–0.8 mg of FA for women of child‐bearing age starting at least one month before conception and continuing through the first 2 to 3 months of pregnancy.^[^ ^5^, ^6^ ^]^ Some comparative data support the use of FA‐containing multivitamins/minerals (MVM), for example, a cohort of 22,776 women who used FA‐containing MVM during the first 6‐weeks of pregnancy showed a decrease in the prevalence of NTDs,^[^ ^7^ ^]^ and another study showed similar reductions by FA alone or FA‐containing MVM relative to no supplementation.^[^ ^1^ ^]^ What is lacking is a more controlled study of the comparative benefits between FA alone and FA‐containing MVM and an experimental context to explore the cellular mechanisms underlying changes in NTD risk.

**Figure 1:**
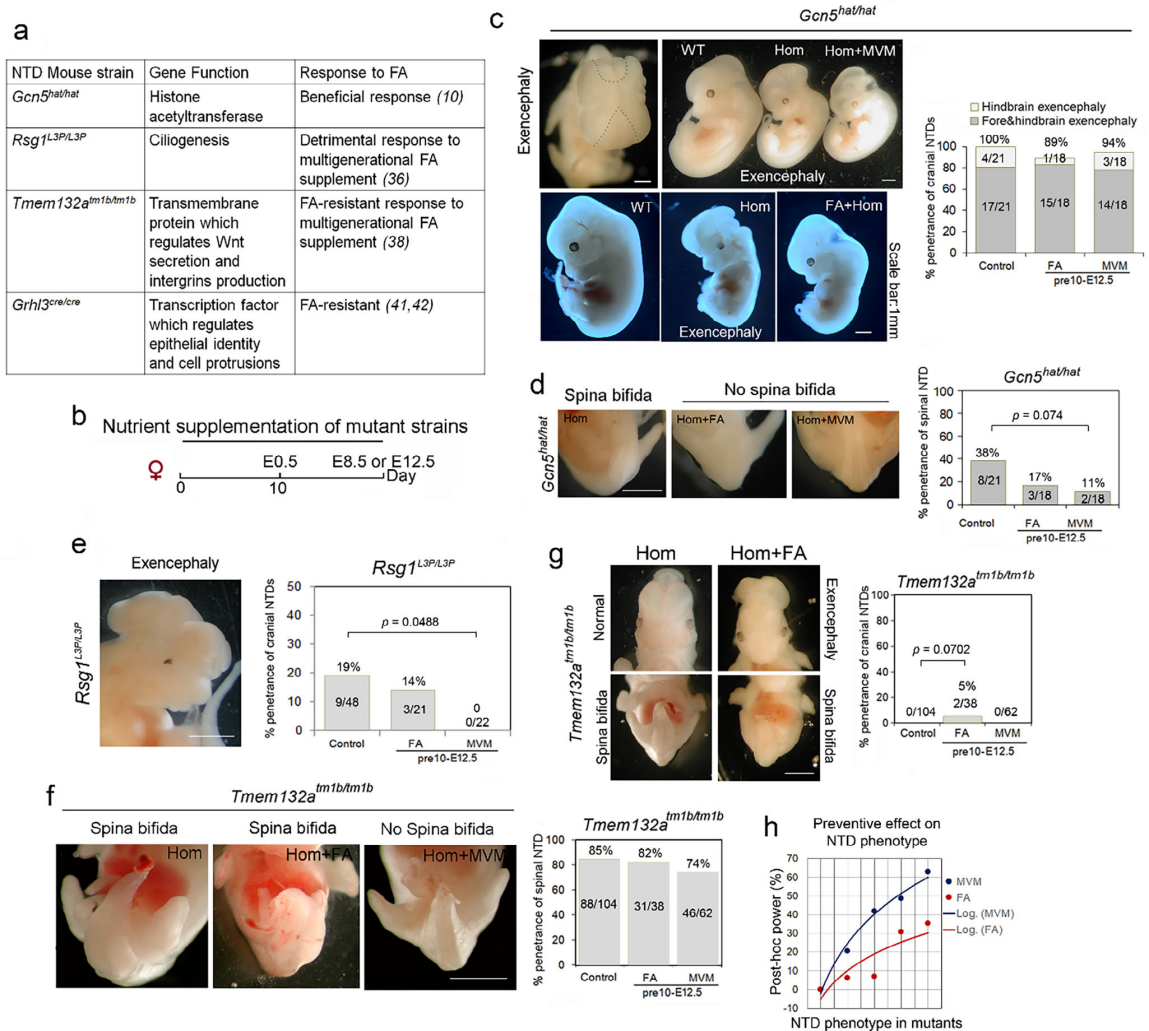
MVM supplementation reduces the incidence and penetrance of neural tube defects. a) Table of mouse gene mutations that cause NTDs and their responsiveness to FA as per references cited. b) Timeline of nutrient supplementation of mouse dams: nutrient supplementation maintained at least 10 days before mating and continued until embryo dissection at E8.5 or E12.5. c) *Gcn5^hat/hat^* mutant embryo showing hindbrain and forebrain exencephaly (left, black dots outline open cranial neural tube) and penetrance of hindbrain and forebrain exencephaly or hindbrain exencephaly at E12.5, following control diet or nutrient supplementation. d) Effect of nutrient supplements on the incidence of sacral spina bifida in *Gcn5^hat/hat^* mutant embryos. e) *Rsg1^L3P/L3P^* mutants with exencephaly and complete rescue of NTD with MVM supplementation. f) *Tmem132a^tm1b/tm1b^* embryos show ~85% sacral spina bifida, and this incidence is reduced by MVM. FA alone increases the risk for cranial NTD g) and craniofacial abnormalities (Figure S2a, Supporting Information). h) Scatter plots and logarithmic trendline show the effects of nutrient supplements on NTD phenotypes in the mutant strains. Positive numbers indicate beneficial effects relative to the control diet. In (c–g), the numbers in the columns represent the number of embryos analyzed, and the penetrance of the phenotype is indicated by the percentage at the top of each column. Fisher's exact test was used and *p* <0.05 was considered as statistical significance. Scale bar: 1 mm.

Animal models, particularly mice, provide a means to explore the genetic causes of NTDs and to couple this with a study of dietary effects on NTD incidence. Mouse genetic studies have identified ∼300 genes involved in the process of neural tube closure and deficiency of these genes leads to NTDs resembling human phenotypes.^[^ ^8^, ^9^ ^]^ Only a few of these mouse models have been tested for responsiveness to dietary factors, and this has resulted in mixed results. Some genetic mutants show a beneficial response to FA (decreased NTD risk). For example, FA can reduce the penetrance of exencephaly in *Gcn5* hypomorphic mutants from 100% to 69%.^[^ ^10^ ^]^ However, other mutants show no response (FA‐resistant) or even a detrimental response (increased risk) including strains listed in Figure 1a.^[^ ^8^ ^]^ Inositol is another crucial nutrient for neural tube closure and its supplementation can rescue NTDs in the FA‐resistant *curly‐tail* mouse model, which is a hypomorphic allele of the *Grhl3* gene.^[^ ^11^ ^]^ Mouse studies have also shown the importance of genes encoding enzymes functioning in vitamin A,^[^ ^12^, ^13^ ^]^ zinc,^[^ ^14^ ^]^ iron,^[^ ^15^ ^]^ and calcium^[^ ^16^, ^17^ ^]^ metabolism in neural tube closure. Moreover, an imbalance of micronutrients such as vitamin C, B12, manganese, selenium, and zinc are associated with an increased prevalence of NTDs in humans^[^ ^18^, ^19^, ^20^, ^21^ ^]^ and maternal intake of micronutrients is associated with a decreased risk of NTDs.^[^ ^20^ ^]^ Here we asked whether MVM supplementation could be more effective in decreasing the prevalence of NTDs. We tested genetically susceptible mouse mutant lines that respond beneficially or detrimentally or not at all to FA alone and found that MVM supplementation is as effective, and in many cases more effective, than FA alone in reducing the penetrance of NTDs.

Human iPSC‐derived neural tube‐like organoids have been reported^[^ ^22^, ^23^, ^24^, ^25^, ^26^, ^27^, ^28^, ^29^, ^30^, ^31^ ^]^ and provide another model to study genetic and nutritional effects. Through application of morphogens and self‐organization, these organoids recapitulate patterning of the neuroectoderm with an adjacent neural plate border^[^ ^22^ ^]^ or features of anterior‐posterior patterning and dorsal‐ventral patterning^[^ ^23^, ^28^ ^]^ or formation of a bi‐layered neuroectoderm and non‐neuroectoderm organoid that mimics neural plate folding and neural fold fusion.^[^ ^25^ ^]^ The biophysical parameters that control morphogenesis into a polarized neuroepithelium have also been studied in organoids.^[^ ^24^ ^]^ These models are exciting and useful, yet none of the organoid systems mimic the elliptical shape of the human neural tube. This could limit their representation of the macroscale cytoarchitecture and cellular positioning.

We developed and characterized human iPSC‐derived 3D neural ectoderm organoids. Although they do not undergo neural plate bending and fusion, the organoids form a lumen and an elongated shape that resembles the shape of the human neural tube. We used this organoid system to evaluate the effect of MVM vs FA alone media on cellular behaviors. FA‐containing MVM supplementation balances decreased apical F‐actin induced by ROCK inhibition and protects the lumen shape. In *GCN5*ΔHAT deletion organoids, MVM rescues the abnormal increase in asymmetrical cell divisions, and does not elicit abnormalities in mitosis seen upon FA alone. High‐performance liquid chromatography (HPLC) analytical assays of organoids and mouse embryos with wildtype genotypes indicate that FA alone results in variable levels of nucleotide metabolites whereas MVM supplementation maintains a more normal balance of metabolites and levels of DNA synthesis and mitosis. Together our studies provide experimental evidence for the increased effectiveness of MVM supplementation in preventing NTDs.

## 2 Results

### 2.1 Establishment of Supplementation Regime in Mice and Assessment of Intracellular Metabolic Levels in Wildtype Mouse Embryos

To test the efficacy of FA‐containing MVM supplementation, we assessed the components of MVM prenatal vitamins ranked in the top four on the Amazon marketplace and compared to the FDA daily nutrition requirements for pregnant and lactating people (http://federalregister.gov/a/2016‐11867). Many of the supplements have adopted ∼100% daily value of the FDA requirements, effectively doubling the nutrients, although levels of individual vitamins or minerals can vary between manufacturers by ∼10 fold (Table S1, Supporting Information). Herein, to model MVM prenatal supplementation in humans, C57BL/6J mice were given regular daily chow ad libitum (Teklad Global 18% protein rodent diet) and were supplemented through drinking water with the same strategy (100% daily value of vitamins and minerals of daily intake in the chow; Table S2 (Supporting Information), their daily consumption of water = ~4.83 g water per pregnant mouse). MVM based on the mouse chow contained Folic acid (FA), Niacin, Vitamin A (Va), Vitamin E, Vitamin D, Vitamin B1 (B1), Vitamin B2 (B2), Vitamin B5 (B5), Vitamin B6 (B6), Vitamin B12 (B12), Biotin, Calcium (Ca), Choline, Copper (Cu), Inositol (INO), Iodine, Iron (Fe), Manganese (Mn), Selenium (Se), Zinc (Zn) (Table S2, Supporting Information). As an initial study, supplementation was provided to both male and female wildtype C57BL/6J breeding age mice for at least 35 days prior to mating (spermatogenesis occurs over 35 days whereas fertilizable oocytes are produced in 4–5 days)^[^ ^32^, ^33^ ^]^ and continued supplementation of the dam until collection and evaluation of the embryos at embryonic day (E)12.5 (Figure S1a, Supporting Information). No gross changes in wildtype embryo morphology were seen (Figure S1b,c, Supporting Information).

As the recommendation is for women to take supplements at least one month before pregnancy (fertilizable oocytes produced in ~30 days), the remainder of the mouse experiments were performed by treating only female mice and decreasing the timing of nutrient supplementation to at least 10 days before mating and continuing until the day of embryo collection (Figure 1b). To assess the impacts of nutrients on metabolites during neurulation, we performed HPLC analytic assays on whole mouse embryos of 6–8 somite stage, at the early stages of neural tube closure. We examined intracellular levels of vitamins, minerals, one carbon metabolites, and amino acids (Figure S1d–g, Supporting Information). FA alone, but not MVM which also contains the same level of FA, robustly elevated intracellular folate levels (Figure S1d, Supporting Information). Consistent with increased folate levels, FA alone increased the level of cystathionine (Figure S1f, Supporting Information), which is an intermediate in the synthesis of cysteine from homocysteine. FA alone or MVM reduced the level of calcium (Figure S1e, Supporting Information), and vitamin B3 niacin was reduced by MVM (Figure S1d, Supporting Information), even though MVM contains these micronutrients. FA alone upregulated levels of L‐asparagine and L‐glutamate and overall showed more variable levels of amino acids than MVM supplementation (Figure S1g, Supporting Information right panels FA vs CON and MVM vs CON). Overall at the onset of neurulation, MVM appears to maintain more stable homeostasis of metabolites than FA alone in wildtype embryos.

### 2.2 MVM Supplementation Decreases Penetrance of NTDs Better than FA Alone in Mouse Genetic Models

To test the efficacy of FA‐containing MVM supplementation vs FA alone on the prevalence of NTDs, we analyzed four mouse models with genetic susceptibility to NTDs (Figure 1a). *GCN5*/*KAT2A* encodes an acetyltransferase. Putatively damaging rare variants are detected in the *KAT2A* gene in NTD cases.^[^ ^34^ ^]^ Deletion of the histone acetyltransferase (HAT) domain in mice (*Gcn5^hat/hat^*) causes 100% penetrant exencephaly on a control diet; and supplementation with high levels of FA (100 µg/30 g body weight, equivalent to 3.33 mg kg^−1^ body weight per day over E0.5–E9.5) reduced exencephaly penetrance by 31%.^[^ ^10^ ^]^ In the present study, at the lower FA alone dose (0.45 mg kg^−1^ body weight per day) or MVM supplementation used to model the dosage in prenatal multivitamins in humans, a non‐significant but biologically relevant reduction in exencephaly penetrance was observed (FA alone: (89% (16/18)); MVM: (94% (17/18)) (Figure 1c). Spina bifida is incompletely penetrant in *Gcn5^hat/hat^* mutants and both FA alone and MVM decreased the incidence of spina bifida (Figure 1d; Control: 38% (8/21); FA alone: 17% (3/18); MVM: 11% (2/18) (*p* = 0.074 vs Control; Fisher's exact test). Thus, in a FA responsive mouse NTD model, MVM treatment was approximately equivalent to FA alone treatment.

Next, we expanded the in vivo observations of the impact of nutrient supplements to test whether MVM was more effective in preventing NTDs in FA non‐responsive or FA‐detrimental mouse genetic NTD models. *Rsg1^L3P/L3P^* cilia mutants^[^ ^35^ ^]^ show a detrimental NTD response to a multigenerational FA diet and a FA resistant response to a short‐term FA diet (from fertilization to dissection) (Figure 1a).^[^ ^36^ ^]^ With MVM supplementation, exencephaly in *Rsg1^L3P/L3P^* mutants was significantly decreased to 0% (0/22) (*p* = 0.0488; Fisher's exact test), whereas the control diet and FA alone diet showed a higher penetrance (19% (9/48) and 14% (3/21) respectively) (Figure 1e). *Tmem132a^tm1b/tm1b^* mutants, in which WNT and Integrin signaling is disrupted,^[^ ^37^, ^38^ ^]^ show incompletely penetrant sacral spina bifida (~85%, Figure 1a,f) and they are resistant to a multigenerational FA diet.^[^ ^38^ ^]^ Our study indicates that MVM supplementation of *Tmem132a^tm1b/tm1b^* mutants slightly decreased the incidence of spina bifida relative to control or FA alone (Figure 1f; MVM: 74% (46/62); Control: 85% (88/104); FA alone: 82% (31/38)). Unexpectedly, FA alone resulted in exencephaly in two *Tmem132a^tm1b/tm1b^* mutants (5% (2/38); *p* = 0.0702; Fisher's exact test) and severe craniofacial malformations in four *Tmem132a* ^tm1b/tm1b^ mutants, which did not occur with control or MVM diets (Figure 1g; Figure S2a, Supporting Information; 11% (4/38) for FA; vs CON: *p* = 0.0045; vs MVM: *p* = 0.0188). *GRHL3*, which encodes a transcription factor required for epithelial identity and cell protrusions, is required for caudal neural tube closure (Figure 1a).^[^ ^39^, ^40^ ^]^ A hypomorphic allele of *Grhl3* called *curly tail* is FA‐resistant but partially responsive to inositol, while the *Grhl3* null mutant is resistant to either FA or inositol.^[^ ^41^, ^42^ ^]^ We tested a *Grhl3^Cre/Cre^* null mutant line and found that the penetrance of spina bifida was decreased from 100% (19/19) to 92% (11/12) when treated with inositol (800 µg g^−1^ body weight per day) from E7.5 to E10.5, but neither FA alone nor MVM prevented spina bifida, although MVM supplementation contains an equivalent dosage of inositol (Figure S2b, Supporting Information).

Finally, we assessed whether supplements could impact other phenotypes in these mutant embryos. *Rsg1^L3P/L3P^* mutants show 100% (48/48) penetrant eye malformations and polydactyl, and *Tmem132a^tm1b/tm1b^* mutants show 100% (104/104) limb symbrachydactyly. These malformations were resistant to MVM or FA alone supplements (Figure S3, Supporting Information). Evaluation of body and placenta weight in *Gcn5^hat/hat^* and *Tmem132a^tm1b/tm1b^* mutants showed a trend toward a negative impact of FA alone whereas MVM showed a trend to a more beneficial effect (Figure S4a–c, Supporting Information). Taken together, although not all malformations respond to nutrient supplementation, MVM appears more beneficial for the prevention of NTDs and other embryonic deficits than FA alone (Figure 1h; Figure S4d, Supporting Information).

### 2.3 Generation of Reproducible Human Neural Tube‐Like Organoids

**Figure 2:**
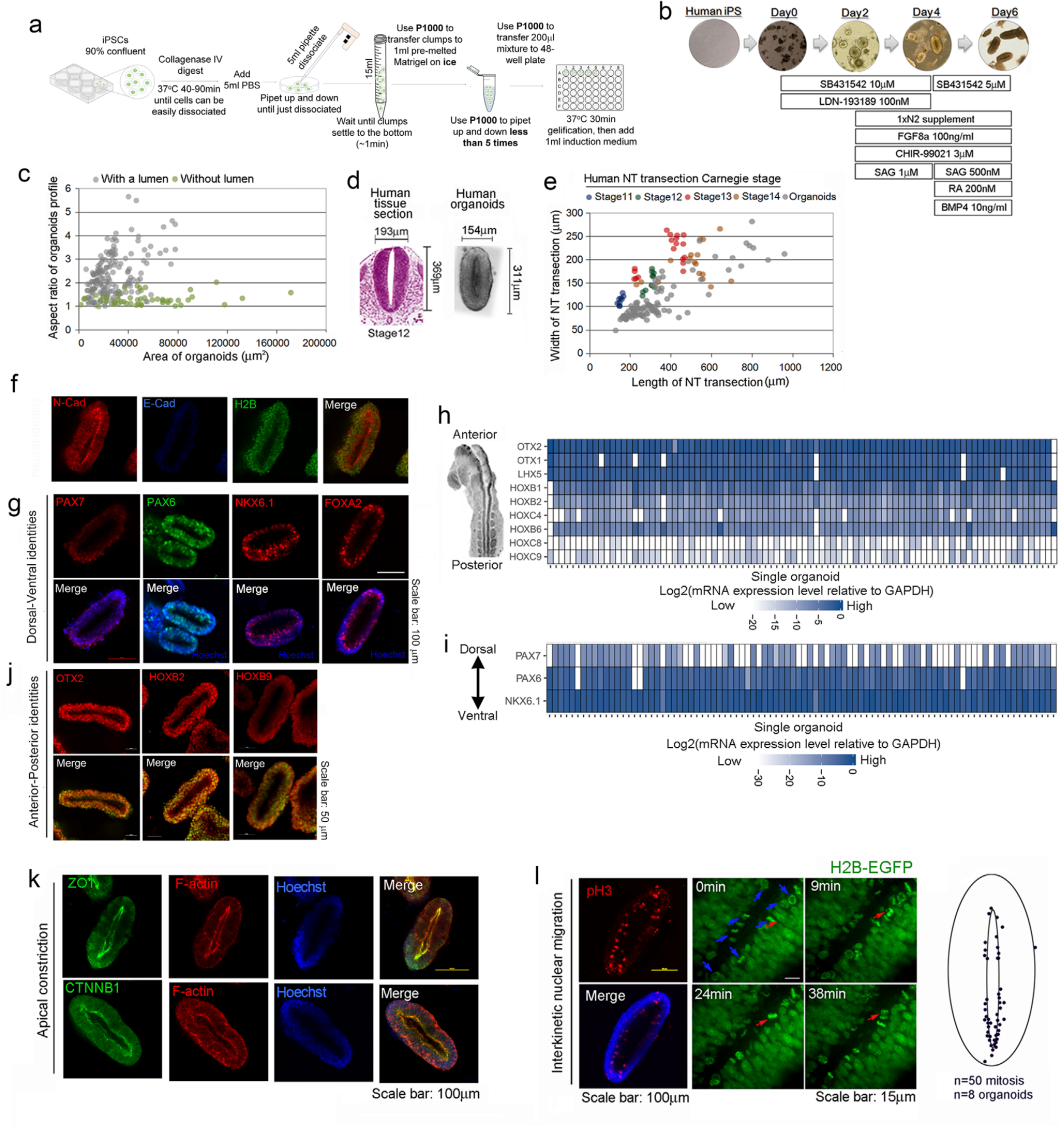
Human iPSC‐derived neural tube‐like organoid generation and characterization. a,b) Schematic of cell‐Matrigel procedure (a) and induction with morphogens (b) to create human iPSC‐derived neuroectoderm organoids. c) Dot plot shows the profile of organoids with a single lumen (gray dots) (*n* = 164) or no lumen (green dots) (*n* = 50). Neural tube organoids with a single lumen in transection are characterized by an area smaller than 80,000 µm^2^ and an aspect ratio (length to width) larger than 1.8. d) Hematoxylin and Eosin‐stained transverse section of a human spinal neural tube at Carnegie stage 12 (left) (From The Virtual Human Embryo (ehd.org)) and representative image of a human neural tube‐like organoid (right) with a similar shape. e) Dot plot comparison of human neural tube‐like organoids (gray dots; *n* = 95; five independent assays) to transverse sections of human spinal neural tube at Carnegie stage 11 (blue dots; 9 sections), 12 (green dots; 11 sections), 13 (red dots; 18 sections) and 14 (brown dots; 17 sections). The measurements of transverse sections of the human spinal neural tube were collected online (https://www.ehd.org/virtual‐human‐embryo/). f,g) Representative fluorescent images of human neural tube‐like organoids for protein expression of (f) neural identity marker N‐cadherin, non‐neural ectoderm marker E‐cadherin, and histone H2B; (g) Dorsal‐Ventral markers PAX7 (dp3‐dp5), PAX6 (dp3‐pMN), NKX6.1 (vp3 to floor plate), FOXA2 (Floor plate); Scale bar: 100µm. h,i) Heatmaps show relative RNA levels of Anterior‐Posterior markers (h) and Dorsal‐Ventral markers (i) in single organoids relative to *GAPDH* (*n* = 90 from three independent experiments). j) Representative fluorescent images for anterior‐posterior markers OTX2 (anterior neural tube marker), HOXB2 (hindbrain) and HOXB9 (posterior neural tube marker). H2B‐EGFP shows nuclei. Scale bar: 50µm. k) Representative fluorescent images for apical constriction markers ZO1 (tight junction) or CTNNB1 (adherens junction) (green), and F‐actin (red), nuclei stained with Hoechst. Scale bar: 100µm. l) Phosphorylated‐H3 (pH3) staining shows mitotic cells are largely distributed near the apical lumen. The middle panels are still images of time‐lapse movies using the H2B‐EGFP cell line to dynamically follow mitosis in organoids. The red arrows in four images trace a single cell undergoing division. Blue arrows at time 0 point out all cell divisions in the field. The right panel is a schematic of the location of mitosis within the human neural tube‐like organoids (50 mitosis of 8 organoids in four independent assays). The scale bar is 100 µm in the left panel and 15 µm in the middle two panels.

To test the efficacy of MVM supplementation vs FA alone in human neural tube tissue and to model the possible cellular mechanisms, we generated a human neural tube‐like organoid model using a 6‐day induction method (Figure 2a,b; Video S1 and Table S3, Supporting Information). Almost 100% of organoids with regular elliptical shape (an aspect ratio larger than 1.8) autonomously formed a single lumen; and closely resembled the shape of the Carnegie stage 11 to stage 14 human neural tube in transection (Figure 2c–e), although the natural neural tube becomes thinner at the dorsal and ventral aspect, whereas organoids have a more homogeneous thickness. In the following experiments, we focused on organoids with a regular elliptical shape and an aspect ratio larger than 1.8.

These organoids were comprised of neural cells as reflected by N‐Cadherin expression, but not non‐neural ectoderm based on the absence of E‐Cadherin expression (Figure 2f). The pluripotency marker OCT4 decreased by Day 4 with concomitant increase in the expression of neural ectoderm markers N‐Cadherin and PAX6, with full expression on Day 5 and 6 (Figure S5a, Supporting Information). Dorsal‐ventral markers PAX7 (dp3‐dp5), PAX6 (dp3‐pMN), NKX6.1 (vp3 to floor plate), and FOXA2 (floor plate) further classified the organoids as of largely ventral and intermediate identity (Figure 2g; Figure S5b, Supporting Information). We extracted mRNA from 90 single organoids from three independent experiments for real‐time qRT‐PCR analysis. The organoids showed largely anterior, ventral and intermediate character (Figure 2h–j; Figure S5b–d, Supporting Information), with high expression of the cranial neural tube markers *OTX1/OTX2/LHX5* and gradually lower expression of hindbrain (*HOXB1/HOXB2*) and spinal cord (*HOXC4/HOXB6*) markers, as well as high expression of intermediate and ventral markers *PAX6* and *NKX6.1* but lower expression of dorsal marker *PAX7* and posterior marker *HOXB9*. Thus, these organoids are characterized as predominantly of cranial to upper spinal neural tube and ventral‐intermediate identity.

Apical constriction is a critical cell behavior in lumen formation and neural tube closure, and the neural tube lumen and hinge points are marked by apical proteins.^[^ ^43^, ^44^ ^]^ The lumen of the organoids shows markers of apical constriction (F‐actin, tight junction marker ZO‐1, and adherens junction protein CTNNB1; Figure 2k). The distribution of CTNNB1 along the apical‐basal surfaces of the cells suggests a pseudostratified neuroepithelium (Figure S5e, Supporting Information). FOXA2 expression tends to be higher in the hinge point regions relative to non‐hinge point regions (Figure 2g; Figure S5f, Supporting Information), consistent with the role for FOXA2 in promoting medial hinge point formation during neurulation.^[^ ^45^ ^]^ Interkinetic nuclear migration (IKNM) throughout the cell cycle is an essential process in maintaining tissue integrity within the pseudostratified neuroepithelium^[^ ^46^ ^]^ and in neural plate bending during neural tube closure,^[^ ^47^ ^]^ with mitosis occurring near the apical surface. Live imaging of organoids created from iPSCs with a H2B‐EGFP allele shows apically localized mitotic divisions, consistent with the apical distribution of the mitotic marker pH3 at the apical endfoot (Figure 2l; Video S2, Supporting Information). These data indicate the usefulness of the organoids in modeling the cellular behaviors of IKNM observed in animal models.^[^ ^44^ ^]^ Collectively, the organoids model the shape of the human neural tube, they are characterized by anterior to upper spinal neural tube and ventral‐intermediate identity, and also provide a suitable model to evaluate apical constriction, IKNM and hinge points which are essential to neural tube closure.^[^ ^43^, ^44^, ^45^, ^48^ ^]^

### 2.4 FA or MVM Supplementation Protects against Defective Apical F‐Actin Distribution

**Figure 3:**
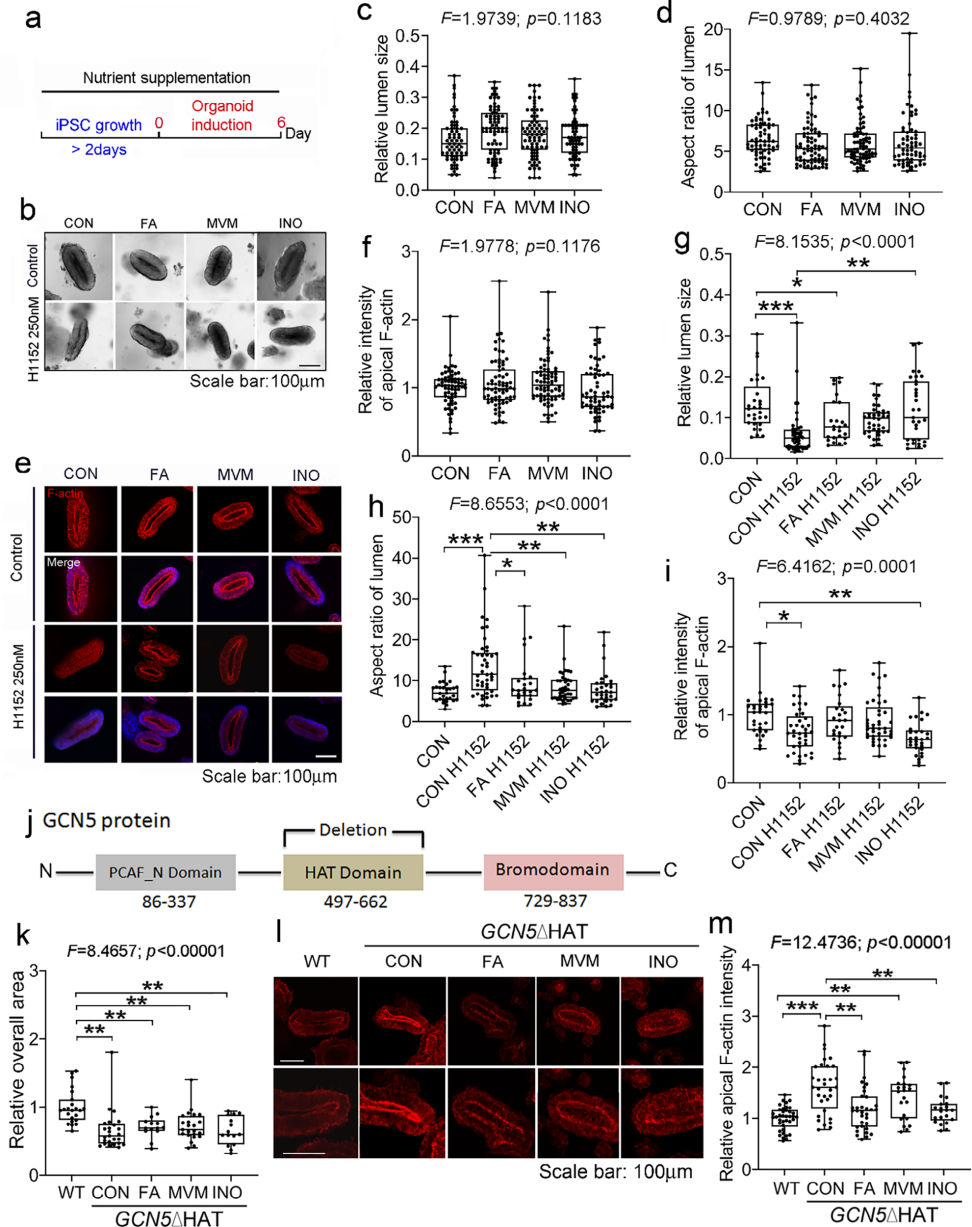
MVM or FA supplementation ameliorates defects in apical F‐actin distribution and lumen formation. a) Schematic timeline of nutrient supplementation during human iPSC growth (>2 days) and organoid induction (6 days). b) Representative bright field images of organoids grown in nutrient supplements without (upper panels) or with (lower panels) ROCK inhibitor H1152. c,d) Quantification of relative lumen size to overall size of organoids (c) or aspect ratio of the lumen (d) in organoids without ROCK inhibitor in control media (CON: *n* = 62); or supplemented with FA (*n* = 71); MVM (*n* = 73); or INO (*n* = 62) from eight independent experiments. e) Representative fluorescent images of apical F‐actin (Phalloidin, red) in organoids grown in control or nutrient‐supplemented media without (upper panels) or with (lower panels) ROCK inhibitor treatment. All images merged with Hoechst (blue). f,i) Quantification of relative mean fluorescent intensity of F‐actin at the apical surface without ROCK inhibitor (f: CON: *n* = 67; FA: *n* = 73; MVM: *n* = 76; INO: *n* = 61) or with ROCK inhibitor (i: CON: *n* = 30; CON H1152: *n* = 37; FA H1152: *n* = 25; MVM H1152: *n* = 41; INO H1152: *n* = 29) from more than four independent experiments. The values are relative to the average of the control. g,h) Quantification of relative lumen size (g) and aspect ratio of lumen (h) of organoids co‐treated with ROCK inhibitor H1152 from four independent experiments (CON: *n* = 29; CON H1152: *n* = 44; FA H1152: *n* = 25; MVM H1152: *n* = 39; INO H1152: *n* = 31). j) Schematic of the protein domains of GCN5 (ENST00000225916.10) and deletion of the HAT domain using CRISPR‐Cas9. k) Box plots show the overall area of *GCN5*ΔHAT mutant organoids grown in control or nutrient‐supplemented media relative to wildtype (WT) organoids in control media (WT: *n* = 23; CON: *n* = 25; FA: *n* = 14; MVM: *n* = 23; INO: *n* = 15 from three independent assays). l,m) Representative images of immunofluorescent staining (l) and quantification (m) of relative F‐actin intensity at the apical surface in wildtype (WT; *n* = 38) or *GCN5*ΔHAT mutant organoids with supplements (CON: *n* = 32; FA: *n* = 33; MVM: *n* = 22; INO: *n* = 21). Data from six independent experiments. Box plots and whisker with all points shown. One‐way ANOVA followed by Post Hoc Tukey HSD. Statistically significant *p* values are denoted ^*^ *p* < 0.05; ^**^ *p* < 0.01; ^***^ *p* < 0.0001. WT: wildtype; CON: Control media; FA: Folic acid; MVM: Multivitamins /minerals; INO: Inositol.

To assess the ability of the human organoid system to model the cellular effects of supplements, we used the following supplementation scheme. Similar to the design for the mouse models, we added the same dosages of vitamins and minerals supplements as the components in the regular iPS cell culture medium (concentrations as in Table S4, Supporting Information, added to the culture media and changed daily, timeline as shown in Figure 3a). This resulted in a final FA concentration equal to ∽10 µm (4.7 µm in regular iPS cell mTeSR1 culture medium plus 4.7 µm supplementation). We acknowledge that this is higher than folate concentrations in human serum which range from 4.5 to 45.3 nm.^[^ ^49^ ^]^ The reasons for this higher concentration are as follows: 1) Standard cell culture medium for all mammalian cells, including iPS cells, is ∽4.7 µm FA and this maintains cells in a healthy state; 2) In human red blood cells, the folate concentration ranges from ∽0.5 to ∽2.3 µm,^[^ ^49^ ^]^ in a similar range as culture medium. It is currently unknown what the local folate concentration is in the developing neural tube; 3) If human serum FA concentration (4.5–45.3 nm) is adopted, the culture system for iPSCs and derived neural tube organoids may then be largely considered as low dosage FA supplementation. The media conditions tested were MVM, FA‐alone or inositol‐alone (same dosage of FA or inositol as in MVM). Supplements did not significantly change the appearance of neural tube organoids (Figure 3, upper panel in b–d) nor did supplements affect the intensity or distribution of apical constriction markers F‐actin and CTNNB1 (Figure 3, upper panels in e,f; Figure S6, Supporting Information). Actomyosin contraction is critical for neural tube closure as disruption of actin turnover and actomyosin disassembly causes forebrain exencephaly and incomplete closure of the posterior neuropore.^[^ ^50^ ^]^ The addition of ROCK‐specific inhibitor H1152 to control media during organoid induction dramatically attenuated apical F‐actin intensity, reduced the lumen size, and increased the aspect ratio, leading to a deformed lumen that was smaller and thinner. Interestingly, MVM or FA alone could protect the shape of the lumen and restore apical F‐actin levels and localization to near control levels (Figure 3b,e,g–i). This is consistent with a report that FA can rescue ROCK inhibition‐induced reduction in apical pMLC intensity and deficiency in apical constriction in MDCK cells and chick neural plate.^[^ ^51^ ^]^ It should be noted that our 3D cultures showed a reduced apical lumen size upon ROCK‐inhibition, whereas the 2D cultures of Tidball et al. 2023 and Takla et al, 2023,^[^ ^52^, ^53^ ^]^ wherein the neural tube organoids are grown on top of the matrix, showed lumen enlargement upon ROCK‐inhibition. Although we have not explored this difference, it may be reflective of a flattening along the z plane in the 2D cultures vs a more symmetric collapse along the x, y, z plane of the lumen in the 3D cultures. Inositol could also prevent disruption of lumen formation by ROCK inhibitor (Figure 3g,h), however, this looks independent on apical F‐actin distribution (Figure 3e,i).

To study the cellular effects of nutrients in the context of a FA‐ and MVM‐responsive NTD associated genetic model, we used CRISPR‐Cas9 gene editing to delete the HAT activity of the GCN5 protein in an iPSC cell line carrying the H2B‐EGFP reporter (Figure 3j; Figure S7a,b, Supporting Information). The HAT deletion made neural induction less successful. In successful inductions, the overall size of *GCN5*ΔHAT organoids were smaller than wildtype (Figure 3k; Figure S7c, Supporting Information). *GCN5*ΔHAT organoids had significantly more accumulation of apical and total F‐actin (CON = control media vs wildtype in Figure 3l,m; Figure S7d, Supporting Information). However, FA alone, inositol alone, and less prominently MVM, could restore F‐actin distribution to more normal levels (Figure 3l,m). Our data collectively indicate that nutrient supplementation can normalize apical F‐actin after pharmacological disruption or genetic deficiency, with implications for supporting in vivo apical constriction and lumen formation during neural tube closure.

### 2.5 MVM or FA Alone Supplementation Prevents *GCN5*ΔHAT‐Induced Premature Neuronal Differentiation

**Figure 4:**
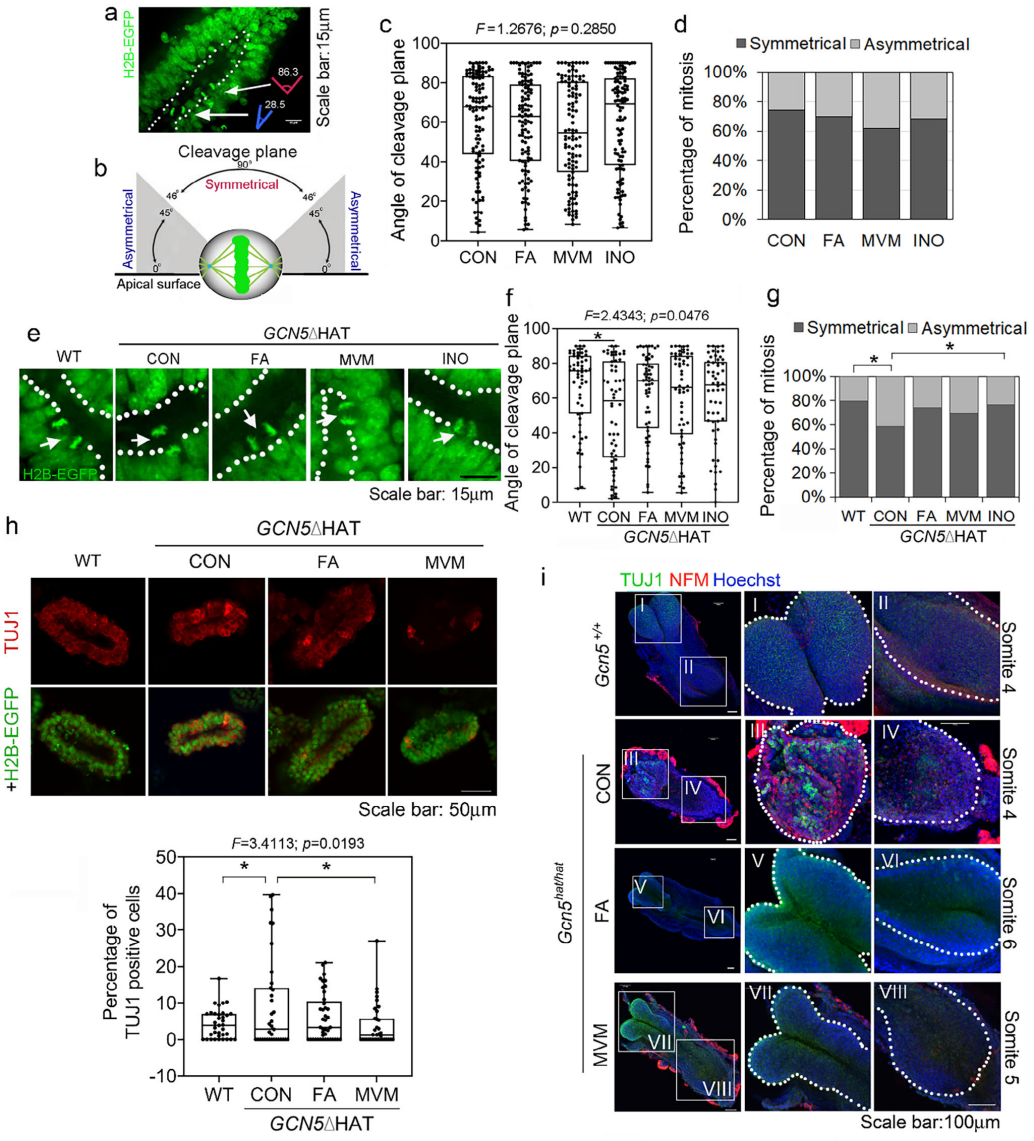
MVM or FA supplementation prevents abnormal asymmetrical cell division and premature neuronal differentiation. a,b) Representative image and schematic to show measurements of angle of cleavage plane during mitosis in H2B‐EGFP iPS cell line‐derived human neural tube‐like organoids. The schematic was modified from.^[^ ^55^ ^]^ c,d) Quantifications of angle of cleavage plane (c) or percentage of mitosis showing symmetrical (dark grey) or asymmetrical (light grey) cell division (d) in control media (CON: *n* = 113) and with nutrient supplements (FA: *n* = 113; MVM: *n* = 111; INO: *n* = 110 from six independent experiments). e) Representative still images of cell division in wildtype (WT) or *GCN5*△HAT mutant organoids. f,g) Quantification of images as in (e) of angle of cleavage plane (f) and percentage of mitosis with asymmetrical cell division or symmetrical division (g) in *GCN5*ΔHAT mutant organoids grown in control or nutrient supplemented media (WT: *n* = 58; CON: *n* = 58; FA: *n* = 58; MVM: *n* = 58; INO: *n* = 56 from four independent assays). h) Representative images (upper panel) and quantification (lower panel) of immunofluorescent staining of neural progenitor marker TUJ1 in wildtype (WT) or *GCN5*ΔHAT mutant organoids grown in control or nutrient‐supplemented media (WT: *n* = 35; CON: *n* = 35; FA: *n* = 41; MVM: *n* = 36 from five independent assays). Scale bar: 50 µm. In (c,f,h), box plots and whiskers with all points shown. One‐way ANOVA followed by Post Hoc Tukey HSD. In (d,g), Fisher's Exact Test was used. ^*^ *p* < 0.05. i) Whole mount immunofluorescent staining of mouse embryos with 4–6 somites from the *Gcn5^hat^* mutant strain with neuronal markers TUJ1 (green) and NFM (red). Hoechst stain shows nuclei. Magnified views of insets show the cranial and caudal regions of the neural tube; dotted lines outline the neuroectoderm. Scale bar: 100 µm. WT: wildtype; CON: Control media; FA: Folic acid; MVM: Multivitamins /minerals; INO: Inositol.

Neural stem cells undergo symmetrical cell divisions to expand their number while asymmetrical cell division generates one daughter cell that perpetuates the stem cell lineage and the other daughter cell that can undergo differentiation.^[^ ^54^ ^]^ Using live imaging of the H2B‐EGFP cell line, we tracked cell division in the human neural tube organoids in control and supplemented media and measured the angle of the cleavage plane during mitosis to evaluate symmetrical and asymmetrical cell division (Figure 4a,b).^[^ ^55^ ^]^ The organoids showed predominantly symmetrical (self‐renewing) division and none of the supplements significantly altered the angle of the cleavage plane or the relative ratios of symmetrical vs asymmetrical cell divisions (Figure 4c,d).

In *GCN5*ΔHAT mutant organoids, there was a significant change in the angle of the cleavage plane with more asymmetrical divisions compared to wildtype (Figure 4e–g). FA and MVM modestly, but inositol markedly, returned the ratio of asymmetrical/symmetrical divisions to more normal levels (Figure 4e–g). As asymmetrical divisions are associated with neuronal differentiation whereas symmetrical divisions are associated with neural progenitor self‐renewal, this suggests that supplements may act to prevent premature differentiation in some susceptible backgrounds. To test this possibility, we examined neuronal differentiation marker TUJ1 in organoids by immunofluorescence. In *GCN5*ΔHAT mutants, the number of TUJ1 positive cells increased, whereas MVM significantly reduced the number, and FA showed a similar trend (Figure 4h; Figure S7e, Supporting Information). To verify this idea in vivo, we assessed TUJ1 and another neuronal differentiation marker NFM during neurulation in *Gcn5^hat/hat^* mutant embryos. Consistent with the organoid data, the expression of both neuronal markers unexpectedly emerged in homozygous mutants at early neural tube stages (4–6 somite stage), whereas FA and MVM supplementation both dramatically suppressed this premature neurogenesis (Figure 4i).

### 2.6 FA Alone Elicits Abnormal Mitosis and IKNM, which is Mitigated by MVM

**Figure 5:**
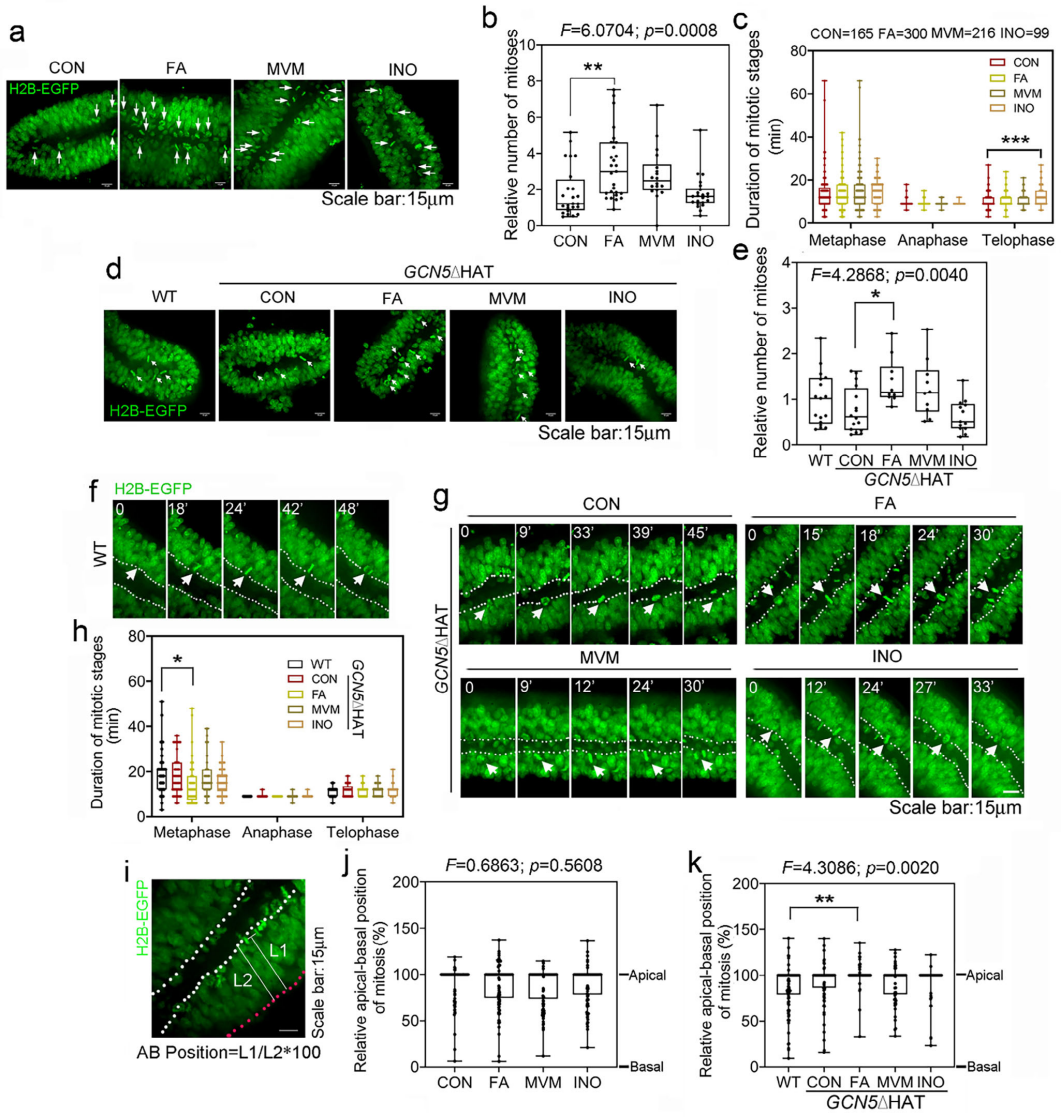
FA elicits abnormal mitosis which can be partially mitigated by MVM supplements. a,b) Representative still images from movies as in Videos S3–S6 (Supporting Information) (a) and quantification (b) of number of mitoses in organoids supplemented with nutrients (CON: *n* = 24; FA: *n* = 27; MVM: *n* = 18; INO: *n* = 22 from six independent assays) using H2B‐EGFP tagged cell line. White arrows indicate all cell divisions at that time point. c) Quantification of duration in minutes of mitotic stages in organoids derived from H2B‐EGFP tagged cell line (CON: *n* =165; FA: *n* = 300; MVM: *n* = 216; INO: *n* = 99 from five independent assays). d,e) Representative still images from movies as in Videos S7–S11 (Supporting Information) (d) and quantification (e) of the number of mitoses in wildtype (WT: *n* = 18) and *GCN5*△HAT mutant organoids with nutrition supplements (CON: *n* = 16; FA: *n* = 10; MVM: *n* = 10; INO: *n* = 13 from four independent assays). f,g) Representative still images of wildtype (f) or *GCN5*△HAT mutant (g) cells transitioning from prophase (panel 1, time point “0”); to the start of metaphase (panel 2); the end of metaphase (panel 3) and start of anaphase (panel 4), and during anaphase (panel 5). h) Quantification of duration of mitotic stages in wild type in control media (WT: *n* = 53) and *GCN5*△HAT mutant organoids grown in control and nutrient‐supplemented media (CON: *n* = 49; FA: *n* = 53; MVM: *n* = 42; INO: *n* = 50 from four independent assays). i) Representative organoid illustrating calculation of apical‐basal position (AB position) as L1 (the length of the shortest distance between the basal surface and the cleavage plane) divided by L2 (the length of the distance between the apical surface and basal surface)*100. j) Quantification of apical‐basal position of mitosis in wild type organoids grown in control or nutrient‐supplemented media (CON: *n* = 113; FA: *n* = 113; MVM: *n* = 111; INO: *n* = 110 from six independent experiments). k) Quantification of apical‐basal position of mitosis in *GCN5*△HAT mutant organoids grown in control or nutrient‐supplemented media (WT: *n* = 95; CON: *n* = 96; FA: *n* = 75; MVM: *n* = 58; INO: *n* = 56 from four independent assays). Box plots and whisker with all points shown. One‐way ANOVA followed by Post Hoc Tukey HSD. ^*^ *p* <0.05; ^**^ *p* <0.01; ^***^ *p* <0.0001. WT: wildtype; CON: Control media; FA: Folic acid; MVM: Multivitamins /minerals; INO: Inositol.

The folate one‐carbon metabolism pathway is key in producing nucleotides necessary for cell division.^[^ ^56^ ^]^ We quantified the rate of cell division normalized to initial cell number, and the duration of each mitotic stage in organoids grown in control or supplemented media (Figure S8a,b, Supporting Information). Live imaging data indicated that relative to control, FA alone significantly increased the number of mitotic cells, whereas MVM (which contains FA) did not cause a significant change and inositol showed no effect on the number of mitoses but increased the duration of telophase (**Figure** **5**a–c; Videos S3–S6, Supporting Information). *GCN5*ΔHAT mutant organoids in control media were similar to wildtype organoids in terms of number of mitoses and duration of each mitotic stage (Figure 5d–h; Figure S8c and Videos S7 and S8, Supporting Information). However, *GCN5*ΔHAT organoids in FA‐alone media had a significantly increased frequency of mitosis (Figure 5d,e; Video S9, Supporting Information), and shortened duration of metaphase (Figure 5f–h). Notably, MVM and inositol maintained both cellular parameters at a level more similar to wildtype organoids with control media (Figure 5d–h; Videos S10 and S11, Supporting Information). Using NE‐4C cells which are derived from mouse E9 cranial neuroectoderm, we also observed that FA alone shortened the duration of metaphase, anaphase and telophase, inositol showed shortened anaphase and telophase, whereas MVM showed mitotic phases similar to wildtype control (Figure S8d,e, Supporting Information).

We next assessed the apical‐basal position of cell division (Figure 5i), which can reflect IKNM activity. For wildtype organoids, supplements caused no significant change in the relative apical‐basal position of cell division (Figure 5j). For *GCN5*ΔHAT mutant organoids, FA alone caused an unexpected shift of cell divisions into the lumen, yet this change was not observed in MVM or inositol alone media (Figure 5g,k). Together these data suggest that MVM helps to overcome FA alone‐induced abnormalities in cell division behaviors.

### 2.7 FA Alone Causes an Imbalance in Nucleotide Metabolites which is Moderated by MVM Supplements

**Figure 6:**
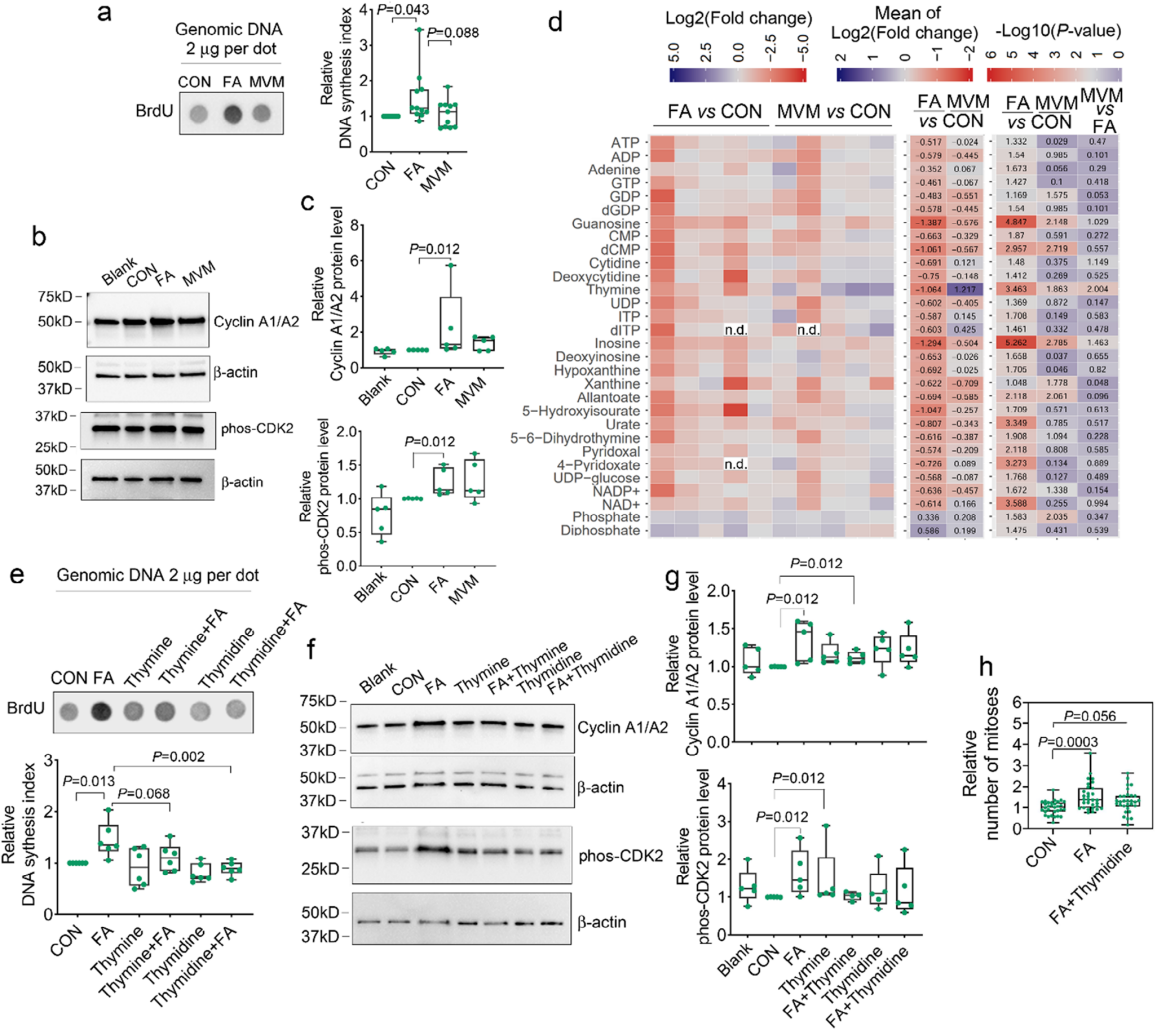
MVM supplementation maintains nucleotide homeostasis and normalizes DNA synthesis. a) Dot blot and quantification of relative levels of BrdU labeled DNA as a reflection of DNA synthesis in organoids with nutritional supplements. *n* = 11 independent biological experiments. b,c) Representative images (b) and quantification (c) of western blot assays show the relative protein levels of S‐phase markers Cyclin A1/A2 and phosphorylated‐CDK2 with FA alone or MVM supplementation. *n* = 5 independent biological experiments. d) Heatmaps show the intracellular levels of nucleotide metabolites represented by individual log2(fold change) relative to control (left panel), mean of log2(fold change) (middle panel) and ‐log10(*P‐*value) (right panel) obtained from HPLC analytics. *n* = 5 independent biological experiments. “n.d.” means not detected. e) Dot blot and quantification of BrdU levels following supplementation of FA alone media with thymine or its derivative thymidine. *n* = 6 independent biological experiments. f,g) Representative images (f) and quantification of western blot assays (g) show relative Cyclin A1/A2 and phosphorylated‐CDK2 protein levels following supplementation of FA alone media with thymine or thymidine. *n* = 5 independent biological experiments. In (b,c,f,g): β‐actin is the reference protein. h) Live imaging in H2B‐EGFP cell line shows relative number of mitoses (CON: *n* = 51; FA: *n* = 47; FA+Thymidine: *n* = 46 from six independent experiments). (a,c,e,g,h): Box plots and whisker with all points shown. (c,e,g): Mann‐Whitney U Test. (d): Student's *t*‐test; two‐tailed. (a,h): one‐way ANOVA followed by Post Hoc Tukey HSD. CON: control; FA: Folic acid. MVM: Multivitamins /minerals.

To uncover the molecular mechanism underlying the ability of MVM to maintain normal cell division parameters whereas FA alone caused cell division abnormalities, we assessed DNA synthesis and nucleotide metabolites. First, we detected BrdU‐labeled DNA synthesis in organoids and found that FA alone significantly enhanced DNA synthesis, while MVM maintained this at a control level (Figure 6a), consistent with the respective findings of the number of mitoses in organoids (Figure 5a,b). Western blot assays against S‐phase cell cycle markers Cyclin A1/A2 and phosphorylated‐CDK2 provided additional support for this observation (Figure 6b,c).

As FA, through the one‐carbon metabolism pathway, transfers a carbon group or methyl group to nucleotide synthesis, we assayed nucleotide metabolites using HPLC analysis. This showed that FA alone in wildtype organoids caused a surprising decrease in purine biosynthesis (ATP, ADP, Adenine, GTP, Guanosine, Inosine) and pyrimidine biosynthesis (dCMP, CMP, Cytidine, Thymine), indicating an overall imbalance in metabolites, relative to control media. In contrast, MVM maintained a more stable homeostasis of these metabolites (Figure 6d; Figure S9a, Supporting Information). To assess this observation in vivo, we assayed nucleotides in wildtype embryos during neurulation (6–8 somite stage). Similar to our observations in human organoids, FA alone elicited upregulation of AMP, GDP and CDP; whereas MVM maintained stable homeostasis of nucleotides although there was a significant decrease in the level of UDP and ADP‐D‐ribose (Figure S9b, Supporting Information).

HPLC analytics of *GCN5*ΔHAT and *Gcn5^hat/hat^* mutants indicated that, relative to wildtype, there is a profound imbalance in nucleotides of both human neural tube‐like organoids and mouse embryos with 6–8 somites (Figure S9c,d, Supporting Information). Purine biosynthesis (Adenosine, ADP, AMP, GMP, GDP, CMP, CDP and IMP) and pyrimidine biosynthesis (dTMP and UMP) are reduced in both *GCN5*/Gcn5 mutant models, suggesting the functional conservation of *GCN5*/*Gcn5* gene and its downstream effectors across humans and mice. However, the salvage pathway of purine synthesis (inosine, hypoxanthine and xanthine) differs in these two models (Figure S9c,d, Supporting Information). Relative to mutants in control conditions, either FA or MVM reduced CMP, 4‐pyridoxate, and NADH and enhanced ADP‐D‐ribose in human organoids (Figure S9c, Supporting Information), but increased guanine and cytidine in mouse embryos (Figure S9d, Supporting Information). This implies that nutrients prevent defects in the *GCN5/Gcn5* mutants likely through differing strategies across mice and humans. Notably, relative to wildtype some metabolites were altered only with FA, such as decreased ATP, cytidine, UTP and NAD+ in human organoids, and increased cytosine in mouse embryos (Figure S9c,d, Supporting Information) and wildtype organoids supplemented with FA alone showed similar alterations of ATP, cytidine and NAD+ (Figure 6d; Figure S9a, Supporting Information), indicating consistent imbalances induced only by FA alone, not genetic mutations. By contrast, MVM maintained a balance of nucleotides more similar to wildtype (Figure S9c,d, Supporting Information). These data indicate a more balanced role of MVM on homeostasis.

To bring additional mechanistic insights into the differences between FA and MVM we focused first on thymine, which was found in inverse relationship in wildtype and *GCN5*△HAT mutant organoids, wherein FA decreased their intracellular levels and MVM increased or made comparable to wildtype levels (Figure 6d; Figure S9a,c, Supporting Information). Therefore, we tested FA alone media supplemented with 2.4 µm thymine or its derivative 0.5 µm thymidine (based on physiological concentrations).^[^ ^57^ ^]^ This supplementation restored the DNA synthesis rate and expression levels of Cyclin A1/A2 and phosphorylated‐CDK2 (Figure 6e–g, Supporting Information) and attenuated the FA‐induced increase in the number of mitoses (Figure 6h; Figure S9e, Supporting Information). Collectively, these data suggest that 1) FA alone leads to an imbalance of the nucleotide pool, whereas MVM maintains homeostasis of the pool. 2) In human organoids, FA alone leads to increased mitosis, whereas supplementation with thymine/thymidine can restore mitosis to normal levels. 3) *GCN5*/*Gcn5* genetic deficit results in many similar downstream effects on nucleotide pools across humans and mice, and although the metabolic responses to nutrients are variable between species, in both cases MVM produces protective metabolic and phenotypic outcomes.

### 2.8 Effective Components in MVM Media that can Alleviate FA Impact on Mitosis

**Figure 7:**
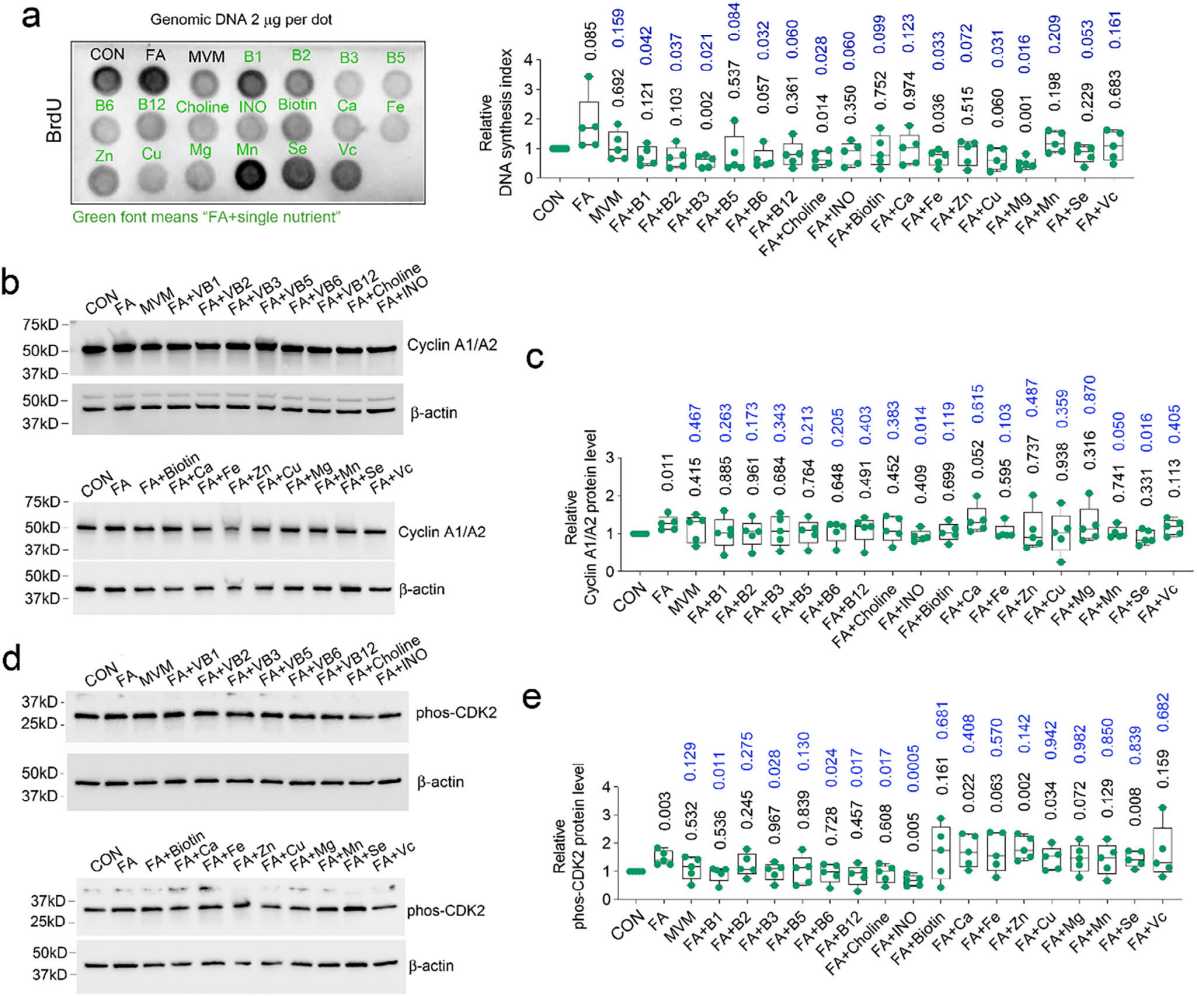
Single nutrients can reverse the increase in mitotic markers induced by FA. Dot blot and quantification of BrdU levels a), Western blot and quantification of relative protein level of Cyclin A1/A2 b,c) and phosphoryated‐CDK2 d,e) following supplementation of FA alone media with single nutrients based on MVM composition. In (a,c,e) the numbers indicate *P* values vs CON (black font) or vs FA (blue font). β‐actin is an endogenous reference protein. Box plots and whisker with all points shown. ANOVA was performed for statistical analysis; *n* = 5 biological replicates. CON: Control; FA: Folic acid; MVM: Multivitamins/minerals.

As MVM media contains multiple minerals and micronutrients, we tested the efficacy of each single nutrient in the context of FA alone media. These single nutrient supplementations did not obviously change the appearance of organoids (Figure S10, Supporting Information). However, Vitamins B1, B2, B3, B6, choline, Fe, Cu and Mg (same concentration as in MVM media) could individually reverse the increase in DNA synthesis induced by FA (Figure 7a). Western blots assays show that vitamin B1, B3, B6, B12, choline and inositol consistently reduced the level of hyperactive phosphorylated‐CDK2 induced by FA whereas inositol or Se significantly decreased the level of Cyclin A1/A2 (Figure 7b–e). Together these results imply that 1) vitamins B can attenuate the increased DNA synthesis induced by FA and 2) the efficacy of MVM is the result of the coordinated action of multiple nutrients.

## 3 Discussion

**Figure 8:**
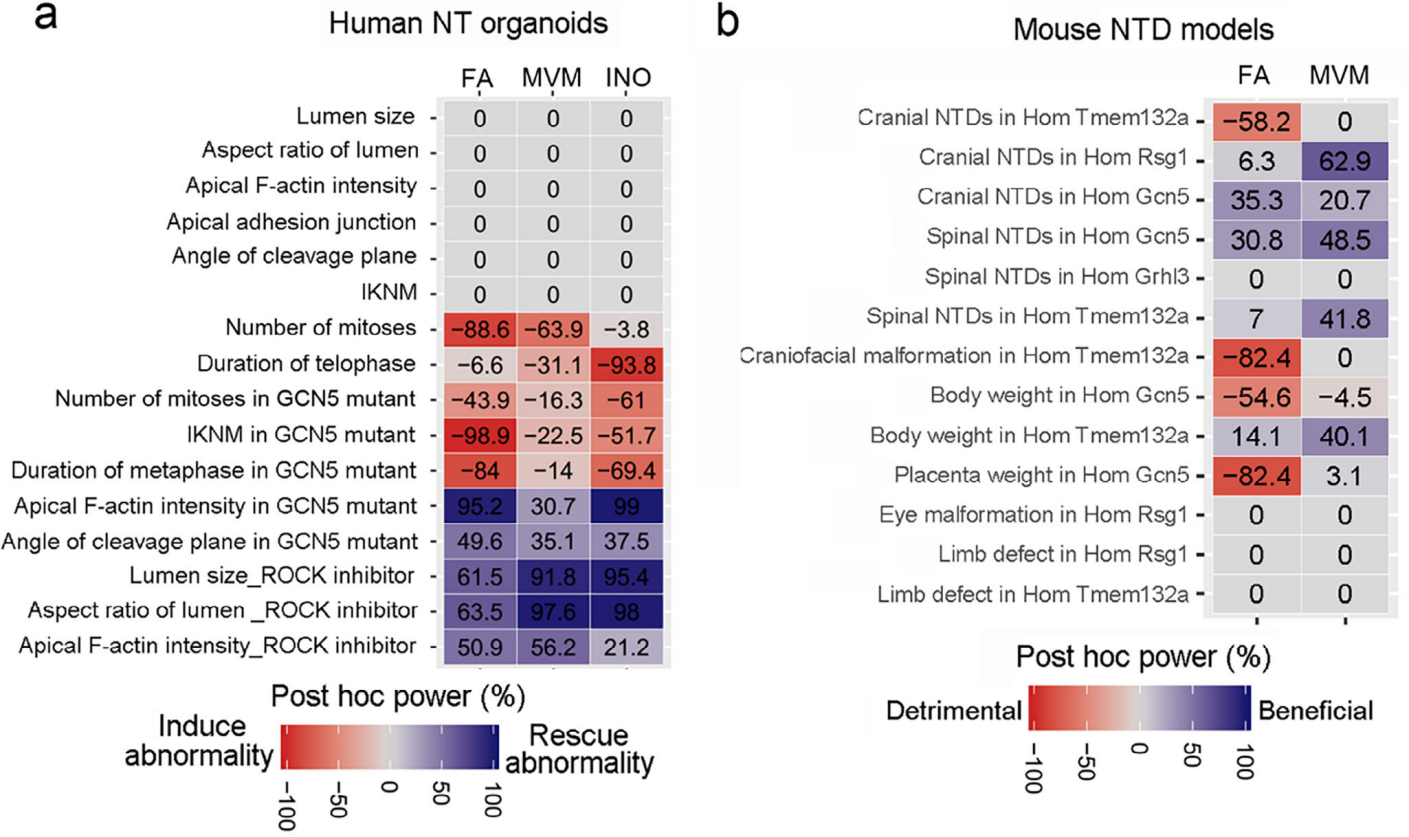
Summary of nutrient impacts on organoid cell behaviors and mouse mutant phenotypes. Heatmaps summarizing data from human neural tube‐like organoids a) and mouse NTD models b). The numbers represent post‐hoc power. “0” means no change between nutrition supplementation and control media or diet.

Our study used human neural tube‐like organoids and mouse genetic models to provide a direct comparison of FA alone vs FA‐containing multivitamin/mineral supplements relative to the cellular behaviors that drive neural tube closure and in the prevention of NTDs. We developed a reproducible human iPSC‐derived neuroectoderm organoid system with features of the neural tube and used this to test how nutrient supplements affect cell behaviors such as organoid shape, F‐actin distribution, and the position and occurrence of cell divisions. Our organoid system provides a powerful medium‐throughput approach to model cellular and molecular mechanisms, with the potential for clinical drug screening. We find that FA‐containing MVM media is more effective than FA‐alone in normalizing cell behaviors following pharmacological or genetic perturbation (Figure 8a). Mouse genetic NTD models also support that MVM supplementation is effective in reducing the incidence of NTDs and in maintaining normal embryonic and placenta growth, and is notable in reducing NTD risk in FA non‐responsive or FA‐detrimental models tested here (Figure 8b). Together our human organoid and mouse NTD data indicate the benefits of a combined supplement of FA‐containing MVM in preventing NTDs. Although further experimentation and evaluation of additional genetic models are necessary to extend this conclusion, our purpose was to lay a framework for future studies to uncover the mechanistic relationship between maternal nutrient supplements and the critical process of neural tube closure.

Previous studies on the mechanisms by which FA decreases the incidence of NTDs in mice have reported 1) modification of apical constriction via MLCK‐dependent activity;^[^ ^51^ ^]^ and 2) promotion of cell cycle progression in *Pax3* mutant neuroepithelium.^[^ ^58^ ^]^ Our data using human neural tube‐like organoids show a protective effect of FA or FA‐containing MVM with respect to apical F‐actin distribution and apical constriction under ROCK inhibitor treatment or *GCN5* HAT domain deletion. A recent paper reported that FA through FOLR1 forms a feedback loop with CD2AP to maintain apical endocytosis and adherens junction complexes,^[^ ^59^ ^]^ which serves to recruit the actin‐binding protein SHROOM3 to the apical cortex to regulate actin distribution.^[^ ^60^, ^61^ ^]^ As apical constriction and SHROOM3 are critical for neural tube closure in both human NTD cases and mouse models,^[^ ^44^, ^61^, ^62^ ^]^ we postulate that one of the key benefits of FA is to balance apical actin distribution through this feedback loop.^[^ ^31^ ^]^ Loss of *GCN5* HAT domain activity in human organoids results in an abnormal increase in asymmetrical cell divisions and premature neuronal differentiation; and both FA and MVM can restore this balance to favor symmetrical cell division and limit premature neuronal differentiation. This finding provided a clue to explore the cellular deficit underlying NTD in *Gcn5^hat/hat^* mouse mutants, which has not been determined.^[^ ^10^, ^63^ ^]^ We observed premature neuronal differentiation in the neural folds of *Gcn5^hat/hat^* mutant mouse embryos, and FA and MVM supplementation suppressed this differentiation during early stages of neural tube closure. As asymmetrical divisions commit neuroepithelial progenitor cells to differentiation, our data, along with the premature differentiation seen in *Pax3* mutants which are also FA‐responsive,^[^ ^58^, ^64^, ^65^ ^]^ provide evidence that FA and MVM can reduce the incidence of NTDs by moderating cell division defects to prevent premature neuronal differentiation.

Human epidemiological and mouse genetic studies show a significant proportion of NTDs are not responsive to FA, and some mouse mutants even show a detrimental response to FA alone. Interestingly, we found that MVM can be beneficial in these cases tested here. MVM completely prevented cranial NTD in *Rsg1^L3P/L3P^* mutants whereas FA alone was only mildly effective in our current study and detrimental upon a multi‐generational FA diet resulting in increased NTD incidence.^[^ ^36^ ^]^ FA alone increased craniofacial defects in *Tmem132a* mutants whereas FA‐containing MVM did not. In human neural tube organoids, FA abnormally increased mitosis and S‐phase proteins and caused aberrant IKNM whereas MVM did not. An increased proliferation rate is associated with an increased risk for NTDs.^[^ ^66^ ^]^ Moreover, nucleotide imbalance can disrupt the coordination of cell growth and cell division.^[^ ^67^ ^]^ HPLC analysis of organoids or wildtype mouse embryos with FA or MVM supplementation showed that FA alone causes an imbalance in intracellular nucleotide metabolites, one carbon metabolism, and amino acid levels whereas MVM maintains more normal levels of these metabolites. Our data further showed that MVM enhances thymine levels in both wildtype and *GCN5* mutant human neural tube‐like organoids. Supplementing FA media with thymine or thymidine can normalize the FA‐induced abnormality in DNA synthesis, the higher level of S‐phase proteins, and the increased incidence of mitosis. Together this data suggests a partial explanation for how MVM can have an increased benefit by balancing key metabolites.

FA alone creates additional imbalances within the cell. Our HPLC analytics of wildtype mouse embryos from FA‐supplemented dams showed variable intracellular levels of folate, calcium, cystathionine, L‐asparagine, L‐glutamate and L‐valine; whereas MVM supplementation resulted in physiological homeostasis of more metabolites. FA supplementation above several hundred micrograms can exceed the metabolic capacity for its reduction and methylation, and this may affect its bioavailability.^[^ ^68^ ^]^ By contrast, vitamin and mineral bioavailability is regulated by homeostatic mechanisms that control absorption or excretion, depending on the nutrient status.^[^ ^69^ ^]^ Epidemiological data indicate an increased risk of NTDs when maternal levels of choline, methionine, vitamin B12, vitamin C, and zinc are low.^[^ ^70^ ^]^ Vitamins can interact with the one carbon metabolism pathway,^[^ ^71^ ^]^ or like zinc, can protect cells from apoptosis to increase the viability of the neuroectoderm.^[^ ^14^ ^]^ MVM provides a broad range of nutrients that can bolster the one carbon metabolism pathway and act through other independent mechanisms, which likely helps to explain why MVM is more effective in decreasing NTD incidence in mouse models with varying response to FA alone (beneficial, resistant, detrimental).

Viewed within the context of the one‐carbon metabolism pathway, B vitamins such as Vitamin B6, function as co‐enzymes of serine hydroxymethyltransferase to transfer tetrahydrofolate to 5,10 methylene‐tetrahydrofolate, the latter which provides a methyl group to dUMP to produce thymidylate (dTMP).^[^ ^56^, ^72^ ^]^ MVM contains vitamin B6 which can help promote biosynthesis of thymidine, and our data shows that thymidine supplementation can reverse the FA‐induced hyperactive cell cycle. A second function of vitamin B6 is its co‐enzymatic role in the removal of homocysteine in the transfer of one carbon units between the folate cycle and methionine cycle.^[^ ^72^, ^73^ ^]^ FA alone supplementation causes an increase of cystathionine, whereas MVM maintains cystathionine at normal levels. In summary, our data show that MVM can counteract the imbalances seen by FA alone supplementation, hence MVM provides a protective benefit. Moreover, the simplified organoid system provides a means to study the cellular‐ and tissue‐level effects of FA, vitamins and minerals on neuroectoderm biology.

Together our cell and animal models help to fill the experimental gap between clinical recommendations of FA supplementation vs FA‐containing multivitamin/mineral supplementation in NTD prevention. While the efficacy of FA supplementation on NTD prevention worldwide should never be neglected, our data highlight the more beneficial effect of combined FA/multivitamin/mineral supplements in balancing critical cellular behaviors of neural tube closure, especially in the context of pharmacological or genetic perturbations. Underlying genetics may influence the response, yet our experimental data overall support the current recommendation of FA‐containing multivitamin/mineral supplements as a better choice than FA alone in preventing birth defects.

## 4 Experimental Section

### Mouse Strains

The C57BL/6J mouse strain was used to study the impact of MVM supplementation on wildtype embryos. The mutant stains *Rsg1^L3P^* (Line3P; from Niswander ENU mutagenesis screen), *Gcn5^hat^* (kind gift from the lab of Sharon Dent), *Grhl3^Cre^* creating a null allele (kind gift from the lab of Shaun Coughlin), and *Tmem132a^tm1b^* *^(KOMP)^* *^Wtsi^* (obtained from UC Davis Knockout Mouse Project Repository) were genotyped according to previously published protocols.^[^ ^36^, ^37^, ^39^, ^63^, ^74^, ^75^ ^]^ *Gcn5^hat^*, *Grhl3^Cre^*, and *Tmem132a^tm1b^* strains were maintained on a C57BL/6J background; *Rsg1^L3P^* was maintained on a 129S1/SvlmJ background. Male breeder mice were used within 1 year of age and all female mice were mated between 8 and 16 weeks of age. Mice were housed at the University of Colorado Boulder in accordance with the Institutional Animal Care and Use Committee (IACUC) approved protocol (Protocol #2659). Embryos were dissected at E12.5 and scored for NTD and other phenotypes characteristic of the line. Embryos of the *Gcn5^hat^* mutant mouse strain were also dissected at E8.5 for following experiments.

### Human iPS Cell Culture and Generation of Neural Tube Organoids

The N2 iPS cell line reprogrammed from dermal fibroblast of a normal human neonate (ATCC: PCS201‐010) was purchased from the Gates Center for Regenerative Medicine at the University of Colorado Anschutz Medical Campus. Mono‐allelic mEGFP‐tagged Hist1H2BJ WTC iPSC line (AICS‐0061‐036) and mono‐allelic mEGFP‐tagged CTNNB1 WTC iPSC line (AICS‐0058‐067) were purchased from the StemTech Center at the University of Colorado Boulder. All iPS cells were maintained in mTeSR1 plus medium (#100‐0276, STEMCELL).

For neural tube organoid generation, a published protocol for human brain region‐specific organoids^[^ ^76^ ^]^ was referred to, and a 6‐day induction medium and culture conditions were created (Figure 2a,b; Table S3, Supporting Information). To initiate differentiation, iPS cells grown in a 6‐well plate until 90% confluence were dissociated by the addition of 1 mL of 2 mg mL^−1^ Collagenase IV (17104019, Gibco) at 37 °C for ∽40–90 min until the cells were easily dissociated but not over‐digested. Cells were immediately dissociated with 5 mL PBS using a 5 mL serological pipette (keeping clump size as large as possible), then transferred to a 15 mL tube. The tube was gently filled up with PBS. The cell clumps were allowed to passively settle to the bottom of the tube (∽1 min, no centrifugation), and the supernatant was gently aspirated leaving <100 µL PBS. Using a P1000 pipette, the cell clumps were transferred into 1 mL pre‐melted Matrigel (354234, Corning, no dilution) on ice. The cell‐Matrigel mixture was fully pipetted up and down no more than 5 times on ice using a P1000 pipette to generate and control the size of cell clumps. 200 µL cell clumps‐Matrigel mixture with ∽5 × 10^5^ cells were transferred into a single well of a 48‐well plate using a P1000 pipette. The plate was incubated at 37 °C for 30 min for gelification, then 1 mL of neural induction media (Day0–Day2) (Table S3, Supporting Information) was added. Media was exchanged every day according to the varied components of the neural induction medium from Day 0 to Day 6 (Table S3, Supporting Information). Organoids were harvested for experiments on day 6.

### Single Organoid Dissociation and Marker Gene Expression Profiling

Many organoids were grown in one 48‐well plate. Single organoids were manually dislodged under a stereo microscope, then the cells transferred with 50 µL PBS into an eppendorf tube. For quantitative real‐time PCR assays for expression of cranial, hindbrain, spinal cord, dorsal to ventral marker genes, total RNA was extracted with TRIzol (Sigma–Aldrich) and finally diluted with DEPC water. Total RNA was used for reverse transcription and cDNA was synthesized using Oligo d(T)_23_VN primer (NEB, #S1327) and First Strand cDNA Synthesis Kit (NEB, #M0253). Quantitative real‐time PCR was performed with the LightCycler 480 Real‐Time PCR System (Roche) against gene primers (Table S5, Supporting Information). Results were normalized to the *GAPDH* gene. Data analysis used the comparative CT method in the Office Excel software.

### Mouse E9 Neuroectoderm Cells

NE‐4C cells (ATCC #CRL‐2925) were derived from E9 mouse embryonic brain. Cells were grown at 37 °C and 5% CO2 under proliferation conditions in MEM (Invitrogen) supplemented with 5% FBS, 1 × MEM nonessential amino acids (Invitrogen) and 1 × GlutaMax (Invitrogen). The NE‐4C cell line was routinely tested in the lab for mycoplasma contamination.

### Nutrient(s) Supplementation or Drug Administration

The dosages of MVM supplements for human iPS cell culture are listed in Table S4 (Supporting Information). MVM supplements in human cell and organoid culture are composed of Biotin, Calcium (Ca), Choline, Copper (Cu), Folic acid (FA), myo‐Inositol (INO), Iron (Fe), Manganese (Mn), Magnesium (Mg), Niacin, Selenium (Se), Vitamin C (Vc), Vitamin B1 (B1), Vitamin B12 (B12), Vitamin B2 (B2), Vitamin B6 (B6), Zinc (Zn) and Vitamin B5 (B5). Each ingredient was separately purchased from Sigma as listed in Table S4 (Supporting Information). The same concentration of FA or inositol was added for FA alone or inositol alone conditions; 0.1% DMSO was the vehicle in Control media (CON). Thymine (2.4 µm) or thymidine (0.5 µm) was supplemented with or without FA. For organoid experiments, all supplements were added for more than two days of human iPS cell culture and throughout the organoid neural induction culture period.

Similarly, for NE‐4C cells, dosages of supplements as in Table S6 (Supporting Information) were used, added to MEM media. Multivitamins/minerals supplements were myo‐Inositol, Calcium, Choline, Folic acid, Vitamin B1, Vitamin B2, Niacin, Vitamin B6. All supplements were added for 48 h before analysis.

For the mouse studies, components were compared according to Teklad Global 18% Protein Rodent Diet and the concentrations as indicated in Table S2 (Supporting Information) were ultimately used. Given the evidence of a beneficial effect of inositol supplements in humans and mice,^[^ ^77^, ^78^ ^]^ inositol (800 µg g^−1^ body weight per day) was added as one component of MVM supplements. Multivitamins/minerals supplements in mice are composed of Biotin, Ca, Choline, Cu, FA, Inositol, Iodine, Fe, Mn, Niacin, Se, Va, B1, B12, B2, B6, Vitamin D, Vitamin E, Zn and B5. Each ingredient was individually purchased from commercial companies as listed in the company columns of Table S2 (Supporting Information). Each single ingredient was weighed and blended as multivitamins/minerals. Supplements were added to the drinking water of mice to minimize injury or stress. The dosages were obtained by calculating nutrients in 2.78 g chow per day as described in the previous paper,^[^ ^36^ ^]^ then added into 4.83 g of drinking water per mouse based on the studies determining the daily consumption of water in the colony. Drinking water was updated every week.

A selective Rho‐kinase (ROCK) inhibitor H1152 (Cat. No. 2414, Tocris) was used to disrupt apical constriction in the organoid system: H1152 (250 nM) was added into the culture medium at Day 5 of induction.

### Immunofluorescent Staining and Confocal Microscopy

Organoids were released from Matrigel using the Corning cell recovery solution (#354253) for 2 h on ice, then fixed for 10 min in 4% PFA, blocked with 1% Triton X‐100 for 1 h at room temperature, and incubated with primary antibody overnight at 4 °C. The primary antibodies used are listed in Table S7 (Supporting Information). Imaging was performed on a Nikon A1 laser scanning confocal microscope. Quantifications were performed in a double‐blinded way and used tools within ImageJ 1.52a.

### Live Imaging

In human organoids, mono‐allelic mEGFP‐tagged Hist1H2BJ WTC iPSC line (AICS‐0061‐036) was used to generate neural tube organoids for live imaging. The cell‐Matrigel mixture was placed in a 4‐chamber 35 mm glass‐bottom dish with 20 mm microwell (D35C4‐20‐1.5‐N, Cellvis). The organoids were imaged from day 5 to day 6 on CellVoyager CV1000 spinning disc confocal microscope (Olympus) with a temperature‐ and gas‐controlled incubator (37 °C, 5% CO2). Before imaging, SPY555‐actin (sc202, SpiroChrome) was added into the culture medium and incubated for 1 h at 37 °C. Images were acquired using a 10x or 20x lens with a long working distance and 488 nm laser and 555 nm lasers. The stepheight was 2.5 µm (∽5 slices/time point, depending on the experiment). The time interval for imaging was 3 min. Post‐image processing and measurements were carried out using ImageJ 1.52a.

Similarly, for imaging of NE‐4C cells, SPY555‐Tubulin (SC203, SpiroChrome) and SPY505‐DNA (SC101, SpiroChrome) were added into the culture medium and incubated for 1 h at 37 °C. Images were then acquired using a 20x lens with long working distance and 488 nm laser and 555 nm lasers. The step height was 2.5 µm (∽5 slices/time point). The time interval for imaging was 3 min. Post‐image processing and measurements were carried out using ImageJ 1.52a.

### Number of Mitoses

In human organoids, the number of mitoses was normalized to initial cell numbers*100, then divided by the number of hours assessed (in general 9 h), then standardized to the corresponding control group.

### Plasmids

To express appropriate sgRNAs in human iPS cells for deletion of the HAT domain of *hGCN5*, the pGL3 vector with the U6 promoter was used and co‐transfected with pCAS9 into cells. pGL3‐U6‐sgRNA‐PGK‐puromycin was a gift from Xingxu Huang^[^ ^79^ ^]^ (Addgene plasmid # 51133; http://n2t.net/addgene:51133; RRID: Addgene_51133) and pCas9_GFP was a gift from Kiran Musunuru^[^ ^80^ ^]^ (Addgene plasmid # 44719; http://n2t.net/addgene:44719; RRID: Addgene_44719).

### Genome editing using CRISPR/Cas9

The CRISPR‐Cas9 DNA editing experiment was adapted from.^[^ ^81^, ^82^ ^]^ Briefly, a pair of complementary oligos for each CRISPR targeting sgRNA (Table S8, Supporting Information) was annealed with 5′ overhangs of “ACCG” and “AAAC.” The annealed DNA was inserted into a BsaI‐linearized pGL3 vector with the U6 promoter. SgRNA sequences were designed to target specific genomic sites for *hGCN5* around the region of the HAT domain. Human iPS cells (AICS‐0061‐036) were transfected with Cas9 (0.5 µg) and two sgRNA plasmids (1 µg) by Lipofectamine Stem Reagent (Invitrogen, STEM00001) one day after plating for 0.5 × 10^5^ cells per well in a 24‐well plate. On Day 2 post‐transfection, puromycin (2 µg mL^−1^) was added and cells were cultured for an additional day. After puromycin selection, single colonies of cells were seeded into individual wells of 96 well plates by limiting dilution. Genomic DNA was isolated for genotyping PCR (Table S9, Supporting Information) to screen for the deletion, followed by Sanger sequencing to evaluate the different deletion clones.

### BrdU Quantitation using DNA Dot Blotting

Optimized from a previous study,^[^ ^83^ ^]^ BrdU (10 µm) was added into the induction medium and the culture continued for 2 h before harvesting the organoids. 200 µL organoids and Matrigel mixture was resuspended in lysis buffer (200 µL, final concentration, 15 mm Tris pH 8.0, 10 mm EDTA pH 8.0, 0.5% SDS, 200 µg mL^−1^ RNase A) by pipetting and rapid inversion of the tube, and then incubated for 1 h at 37 °C. Proteinase K was added at a concentration of 200 µg mL^−1^ of lysate and incubated for 10 h at 56 °C. One volume of Tris pH 7.9 saturated phenol:chloroform:isoamyl alcohol (25:24:1) was added to the sample, and the sample thoroughly shaken by hand for ∽30 s, followed by centrifugation at room temperature for 5 min at 13000 × g. The upper aqueous phase was transferred to a fresh tube. An equal volume of chloroform was added to remove the phenol, shaken by hand, recentrifuged, and the top aqueous phase transferred to a fresh tube. Genomic DNA was precipitated by adding 0.1 sample volume of sodium acetate (3 m) pH 8.0 and 2 volumes of 100% ethanol. The precipitated DNA was washed twice with 70% ethanol and suspended in Tris EDTA buffer (10 mm Tris HCl, pH 8.0, 1 mm EDTA).

The isolated DNA (20 µg per sample) was denatured in NaOH (0.1 m) for 10 min at 95 °C. The DNA was neutralized with NH_4_OAc (1 m) on ice, and then diluted to 20 µL. 2 µL (2 µg) of DNA was spotted onto a hybridization transfer membrane (#NEF‐978A, Biotechnology Systems). The DNA was fixed on the membrane at 80 °C for 30 min, then the membrane blocked in 5% fat free milk in TBS‐T for 1 h at room temperature. To visualize the BrdU signal, the membrane was incubated with mouse anti‐BrdU monoclonal antibody (1:500 dilution, MAB3222, Millipore Sigma) at 4 °C overnight, and then incubated with a secondary antibody, HRP‐conjugated goat anti‐mouse immunoglobulin‐G (IgG) (1:2000) in TBS‐T for 1 h at room temperature. The secondary antibody signal was visualized using a chemiluminescence kit according to the manufacturer's instructions. The intensity of each BrdU signal was quantified by ImageJ 1.54f and corrected for the DNA dilution.

### Nucleotide Metabolite Analysis

University of Colorado School of Medicine Metabolomics Core generated the metabolomics data. Metabolites were extracted from weighed (nearest 0.1 mg) frozen organoid specimens using ice‐cold 5:3:2 methanol:acetonitrile:water to a final concentration of 15 mg tissue per mL.^[^ ^84^ ^]^ Mixtures were vortexed 30 min at 4 °C and spin down for 10 min at 18,000 g, 4 °C. Samples were randomized, then 10 µL of extracts were injected into a Thermo Vanquish UHPLC system (San Jose, CA, USA) and resolved on a Kinetex C18 column (150 × 2.1 mm, 1.7 µm, Phenomenex, Torrance, CA, USA) at 450 µL min^−1^ through a 5 min gradient from 0 to 100% B (mobile phases: A = 95% water, 5% acetonitrile, 1 mm ammonium acetate; B = 95% acetonitrile, 5% water, 1 mm ammonium acetate) in negative ion mode.^[^ ^85^ ^]^ Solvent gradient: 0–0.5 min 0% B, 0.5–1.1 min 0–100% B, 1.1–2.75 min hold at 100% B, 2.75‐3 min 100–0% B, 3–5 min hold at 0% B. Injections were then repeated for positive ion mode at 450 µL min^−1^ through a 5 min gradient from 5 to 95% B (mobile phases: A = water, 0.1% formic acid; B = acetonitrile, 0.1% formic acid) in positive ion mode. Solvent gradient: 0–0.5 min 5% B, 0.5–1.1 min 5–95% B, 1.1–2.75 min hold at 95% B, 2.75–3 min 95–5% B, 3–5 min hold at 5% B.^[^ ^85^ ^]^ Eluant was introduced to the mass spectrometer (Thermo Orbitrap Exploris 120) using electrospray ionization. For both negative and positive polarities, signals were recorded at a resolution of 70000 over a scan range of 65–900 m z^−1^. The maximum injection time was 200 ms, microscans 2, automatic gain control (AGC) 3 × 10^6 ions, source voltage 4.0 kV, capillary temperature 320 °C, and sheath gas 45, auxiliary gas 15, and sweep gas 0 (all nitrogen). The resulting raw files were converted to the mzXML format using RawConverter. Metabolites were assigned and peak areas integrated using MAVEN (Princeton University) in conjunction with the KEGG database and an in‐house standard library.^[^ ^84^, ^86^ ^]^

### Amino Acid, One Carbon Metabolism, Vitamins, and Minerals Analysis

Mouse tissue from whole embryos of 6–8 somites were collected. Metabolites were extracted via a modified protein crash as previously described.^[^ ^84^, ^85^, ^87^ ^]^ Extraction of metabolites was as follows: 250 µL of cold MeOH:ACN:H_2_O (5:3:2, v:v:v) was spiked into each embryo sample. Samples were then vortexed at 4 °C for 30 min. Following vortexing, samples were centrifuged at 12,700 rpm for 10 min at 4 °C, and the supernatant was transferred to a new tube and dried down via SpeedVac (Thermo). Samples were then resuspended in 30 µL of 5:95 MeOH:0.1% formic acid. A portion of the extract from each sample was also combined to create a technical mixture, injected throughout the run for quality control.

High‐throughput Metabolomics Analysis were performed as previously published via a modified gradient optimized for the high‐throughput analysis of metabolomics.^[^ ^84^, ^85^, ^87^ ^]^ Briefly, the analytical platform employs a Vanquish UHPLC system (Thermo Fisher Scientific, San Jose, CA, USA) coupled online to an Exploris120 mass spectrometer (Thermo Fisher Scientific, San Jose, CA, USA). Metabolomics extracts were resolved over an ACQUITY UPLC BEH C18 column (2.1 × 100 mm, 1.7 µm particle size) held at 45 °C (Waters, MA, USA). For positive mode mobile phase (A) 0.1% FA in water and mobile phase (B) 0.1% FA in ACN was used. For negative mode mobile phase (A) Ammonium Acetate (10 mm) in Water and mobile phase (B) Ammonium Acetate (10 mm) in 50:50 ACN:MeOH was used. For negative and positive mode analysis the chromatographic gradient was as follows: 0.45 mL min^−1^ flowrate for the entire run, 0% B at 0 min, 0% B at 0.5 min, 100% B at 1.1 min, 100% B at 2.75 min, 0% B at 3 min, 0% B at 5 min. For positive ion mode, the Exploris 120 mass spectrometer (Thermo Fisher) scanned in Full MS mode from 65 to 975 m z^−1^ at 120 000 resolution, with 3.5 kV spray voltage, 50 sheath gas, 10 auxiliary gas. For negative ion mode, the Exploris 120 mass spectrometer (Thermo Fisher) scanned in Full MS mode from 65 to 975 m z^−1^ at 120,000 resolution, with 3.4 kV spray voltage, 50 sheath gas, 10 auxiliary gas. Calibration was performed prior to analysis using the Pierce Positive and Negative Ion Calibration Solutions (Thermo Fisher Scientific).

Acquired data was converted from raw to the mzXML file format using RawConverter (Lunaweb GmbH). Analysis was done using MAVEN, an open‐source software program for metabolomics analysis. Samples were analyzed in randomized order with a technical mixture injected interspersed throughout the run to qualify instrument performance.

### Statistical Analysis

One‐way ANOVA followed by post‐hoc Tukey HSD test or Mann‐Whitney *U* Test, or Student's *t*‐test were used to analyze the experimental data in organoids and continuous variable in embryos. Fisher's exact test was used to calculate *the p‐*value in the data of a dichotomous phenotype in embryos. To evaluate the effect of nutrient supplementation on the various embryonic phenotypes, the post‐hoc power value was calculated. *p* <0.05 was considered statistically significant. For boxplots, midlines represent the median, boxes the interquartile range (25th to 75th percentile), and whiskers with all points. The statistical details can be found in the figure legends. Statistical analysis was performed using SPSS (25.0.0) and GraphPad Prism 9.0.0 (121). GraphPad Prism 9.0.0 (121) or Excel were utilized for data visualization.

## Conflict of Interest

The authors declare no conflict of interest.

## Author Contributions

H.L. and L.A.N. performed conceptualization. H.L. and J.Z. carried out the methodology. H.L., J.Z., and L.B. performed the investigation. H.L. completed visualization. L.A.N. secured funding and provided supervision. H.L. prepared the Writing–original draft. H.L., J.Z., and L.A.N. conducted review and editing.

## Supporting information



Supporting Information

Supporting Information

Supporting Information

Supporting Information

Supporting Information

Supplemental Video 1

Supplemental Video 2

Supplemental Video 3

Supplemental Video 4

Supplemental Video 5

Supplemental Video 6

Supplemental Video 7

Supplemental Video 8

Supplemental Video 9

Supplemental Video 10

Supplemental Video 11

## Data Availability

The data that support the findings of this study are available from the corresponding author upon reasonable request.
