# Supplementary material for: Effectiveness of Multivitamins vs Folic Acid on Prevention of Neural Tube Defects in Mouse Genetic Models and Human Organoids
Source: Adv Sci (Weinh). 2025 Dec 8;13(8):e13609. doi: 10.1002/advs.202513609 (PMC12884776; doi:10.1002/advs.202513609)
Supplement: Supplementary file 1 — Supporting Information [file ADVS-13-e13609-s009.pdf]

Supplementary Information for

Title: Effectiveness of Multivitamins Versus Folic Acid on Prevention of Neural Tube Defects in Mouse Genetic Models and Human Organoids

Author: Huili Li, Jing Zhang, Lori Bulwith, Lee Niswander*

The supplementary information includes Table S3, S5, S7-S9; Fig S1-S10; Captions for Movies S1 to S11.

Table S1, S2, S4, S6, movies S1 to S11 are separately uploaded.

Table S3: Induction media composition

Items	Vendor	Cat. No.	Day0-Day2	Day2-Day4	Day4-Day6
DMEM/F12	Gibco	11320082	32ml	40ml	40ml
20% Knockout serum	Gibco	10828010	8ml		
100× GlutaMAX	Gibco	35050061	400μl	400μl	400μl
100× MEM-NEAA	Gibco	11140050	400μl	400μl	400μl
1000× 2-Meraptoethanol	Gibco	21985023	40μl		
SB431542	Cayman	13031	10nM	10nM	5nM
LDN193189	Stemcell	72147	100nM	100nM	
100× N2-Supplement	Gibco	17502048		400μl	400μl
CHIR99021	Stemcell	72054		3μM	3μM
SAG	Sigma	566660		1μM	500nM
FGF8a	SinoBiological	16124-HNAE		100ng/ml	100ng/ml
BMP4	R&D	314-BP-010			10ng/ml
Retinoic acid	Sigma	R2625			200nM

Table S5: Primers for marker genes in single organoid-qRT-PCR reaction

OTX1+	AAACAACCCCCATACGGCAT
OTX1-	CAGCTGTGAACGCGTGAAG
OTX2+	CTGTTTGCCAAGACCCGGTA
OTX2-	AAACCATACCTGCACCCTCG
LHX5+	TGTGTAACAAGCAGCTGTCCA
LHX5-	GACAAACTGCGGTCCGTACA
PAX6+	AGCCCTCACAAACACCTACAG
PAX6-	TCATAACTCCGCCCATTCACC
HOXB2+	AAATCCGCCAAGAAACCCAGC
HOXB2-	CCATCTGCAGGCGATCCGA
HOXB1+	AGCTACGGGCCTTCTCAGTA
HOXB1-	CTCCGTAGCCATCGGACAAG
HOXC4+	CCACCACCCCGAGAAATCAC
HOXC4-	TTGGGGTTCACCGTGCTAAC
HOXB6+	TCCAGAACCGACGCATGAAGT
HOXB6-	TCCCTTTCCAGCACCTTCACT
HOXC8+	TGAGACCCCACGCTCCG
HOXC8-	TCCCAGGGCATGAGAGACTT
HOXC9+	GACCTGGACCCCAGCAAC
HOXC9-	CAGCGTCTGGTACTTGGTGT
PAX7+	GGAGGATGAAGCGGACAAGA
PAX7-	GCTCCTCGCGGGTGTATATG
NKX6.1+	CCCACTTTTTCCGGACAGCA
NKX6.1-	ACCAGACCTTGACCTGACTCT
GAPDH+	AGCCACATCGCTCAGACAC
GAPDH-	GCCCAATACGACCAAATCC

Table S7: Antibody used in the present study.

Antibodies	Vendor	Cat. No.
Phospho-Histone H3(Ser10)	ThermoFisher	06-570
Goat anti-Rabbit IgG(H+L) cross-adsorbed secondary antibody, Alexa Fluor 488	ThermoFisher	A-11008
Goat anti-Mouse IgG(H+L) cross-adsorbed secondary antibody, Alexa Fluor 488	ThermoFisher	A-32723
N-cadherin	CST	13116T
E-cadherin	CST	14472S
FOXA2	CST	8186T
ZO-1	Fisher	40-2200
PAX6	Biolegend	901301
NKX6.1	CST	54551
PAX7	R&D	MAB1675
OLIG2	NOVUS	NBP1-28667SS
Alexa Fluor 568 Phalloidin	ThermoFisher	A12380
SPY555-actin	SpiroChrome	sc202
SPY555-Tubulin	SpiroChrome	sc203
SPY505-DNA	SpiroChrome	sc101
Cyclin A1/A2	ABclone	A2635
Phospho-CDK2-T160	ABclone	AP0325
β-Actin	CST	4967S
OTX2	Novus	NBP2-37597
HOXB2	ABclone	A20158
HOXB9	ABclone	A10222
TUJ1	Biolegends	802001
NFM	Thermofisher	13-0700
OCT4	R&D	MAB1759-SP

Table S8: sgRNA in CRISPR-Cas9

Sg-hGCN5-5’HAT_f	accgTGGGGATGTCACCCATCACA
Sg- hGCN5-5’HAT_r	aaacTGTGATGGGTGACATCCCCA
Sg-hGCN5-3’HAT_f	accgGGTGAAGTAGGGGACGCAAA
Sg- hGCN5-3’HAT_r	aaacTTTGCGTCCCCTACTTCACC

Table S9: Genotyping primers in *GCN5*ΔHAT mutant of human organoids

HAT_DEL (1) +	GCTCCCTGAGTCTGGATTCT	Tm= 58.51
HAT_DEL (1) -	GAGGGTTCAAACTGTCCCTG	Tm= 58.1

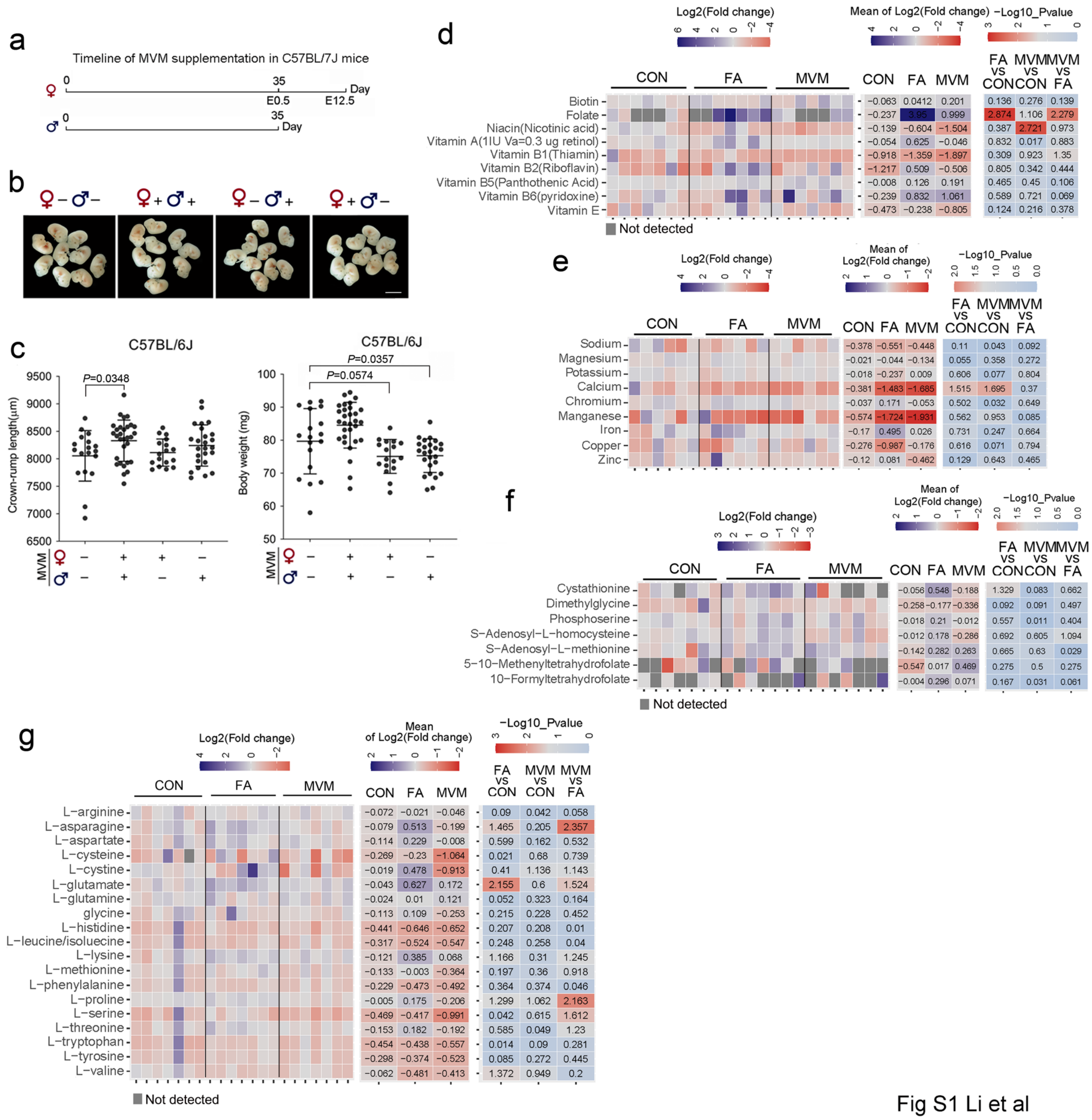

Fig S1 Li et al

Fig. S1: Maternal MVM supplementation minimally impacts early embryo growth and metabolism relative to FA alone supplementation

(**a**) Schematic timeline of multivitamins/minerals (MVM) supplementation of C57BL/6J wildtype mice. (**b**) Representative images show all embryos from individual litters dissected at E12.5 following MVM supplementation provided to female only, or male only, or both. "+" means with supplementation; "-" means without supplementation. Scale bar: 5mm. (**c**) Quantification of crown-rump length and body weight of E12.5 embryos with MVM

supplementation of female dam only (*n* = 17 embryos from 3 litters) or male sire only (*n* = 25 embryos from 3 litters), or both the male sire and female dam (*n* = 30 embryos from 4 litters) relative to control diet (*n* = 18 embryos from 4 litters) (*$p < 0.05$, one-way ANOVA). "+" means with supplementation; "-" means without supplementation. Heatmaps show HPLC analytics of the intracellular levels of vitamins (**d**); minerals (**e**); one carbon metabolites (**f**); and amino acids (**g**) in whole body of C57BL/6J embryos (each column is an individual embryo) at 6-8 somites of age supplemented with CON (control); FA (Folic acid) or MVM (multivitamins/minerals) diets. The data is represented by individual log2(fold change) relative to control (left panel; first the mean for all control embryos was calculated and then the fold change from the control mean was calculated for each individual embryo), mean of log2(fold change) (middle panel) and -log10(*p*-value) (right panel). *n* = 6 or 7 independent individuals. Dark gray blocks in **d**, **f**, **g** mean not detected. The supplementation timeline in (**d-g**) is as Figure. 1b.

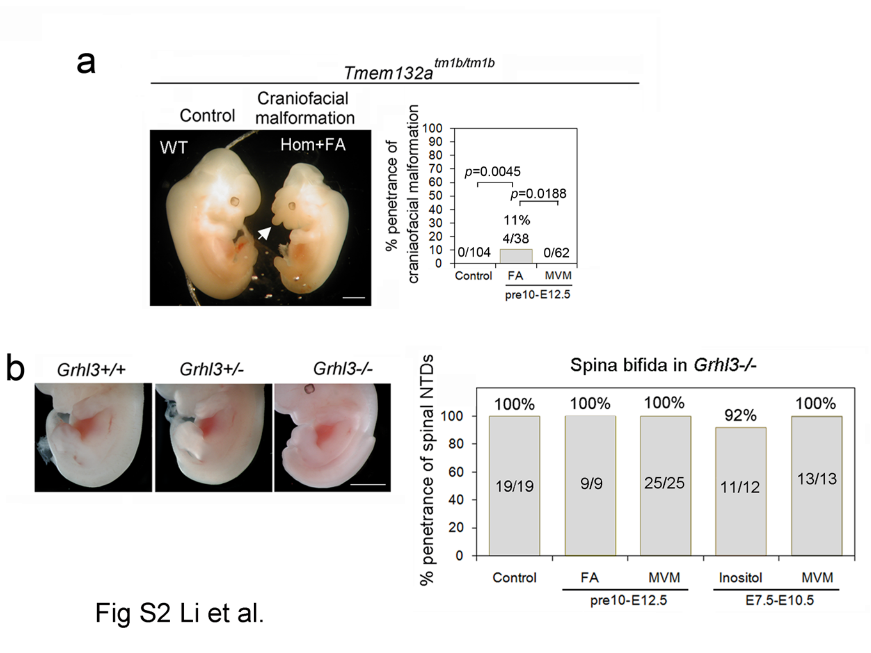

Fig. S2: Impact of supplementation on *Tmem132a$^{tm1b/tm1b}$* mutant and *Grhl3$^{-/-}$* mutant embryos.

(**a**) FA supplementation of *Tmem132a$^{tm1b/tm1b}$* mutants elicits severe craniofacial defects (arrow), which are not observed with FA-containing MVM supplementation. Fisher's exact test was performed. (**b**) The left panels show representative images of spina bifida phenotype in *Grhl3$^{cre/cre}$* (*Grhl3$^{-/-}$*) mutant embryos at E12.5, compared to *Grhl3$^{+/+}$* wildtype and *Grhl3$^{+/-}$* heterozygous littermates. Right panel is quantification of all embryos indicating that neither FA nor MVM can reduce the penetrance of spina bifida in the *Grhl3$^{-/-}$* mouse strain. The numbers above or in the columns means numbers of embryos with phenotypes/total embryos observed. Scale bar: 1mm.

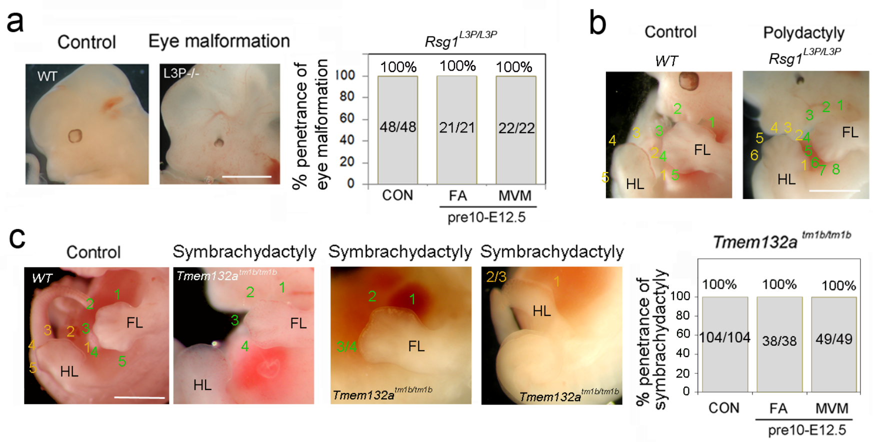

Fig S3 Li et al

Fig. S3. Nutrients do not reduce the penetrance of eye and digit abnormalities.
(**a**) FA or MVM supplementations do not change the 100% incidence of eye defects in *Rsg1*$^{L3P/L3P}$ mutants. (**b-c**) FA or MVM supplementation does not impact incidence of polydactyly in *Rsg1*$^{L3P/L3P}$ (**b**) or symbrachydactyly in *Tmem132a*$^{tm1b/tm1b}$ mutants (**c**). The colored numbers show the number of digits. FL denotes forelimb, HL denotes hindlimb. The numbers above or in the columns means numbers of embryos with phenotypes/total embryos observed. Scale bar: 1mm.

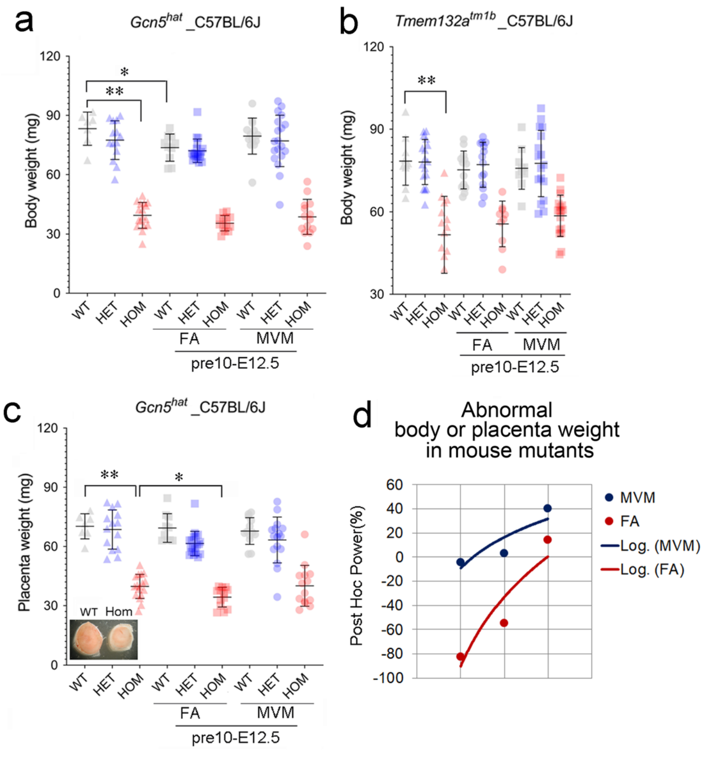

Fig S4 Li et al

Fig. S4. FA alone can induce abnormal body and placenta weight, which can be prevented by multivitamins/minerals supplementation.

(**a-c**) The effects of nutrient supplements on embryonic body weight in *Gcn5^{hat}* (**a**) and *Tmem132a^{tm1b}* (**b**) mutant strains and placental weight in *Gcn5^{hat}* line (**c**). *$p < 0.05$; **$p < 0.01$. One-way ANOVA. (**d**) Scatter plots and logarithmic curve fitting with Post Hoc Power show the effects of nutrient supplements on body or placental weight. Positive numbers indicate beneficial effects; negative numbers indicate detrimental effects. FA: folic acid; MVM: multivitamins/minerals.

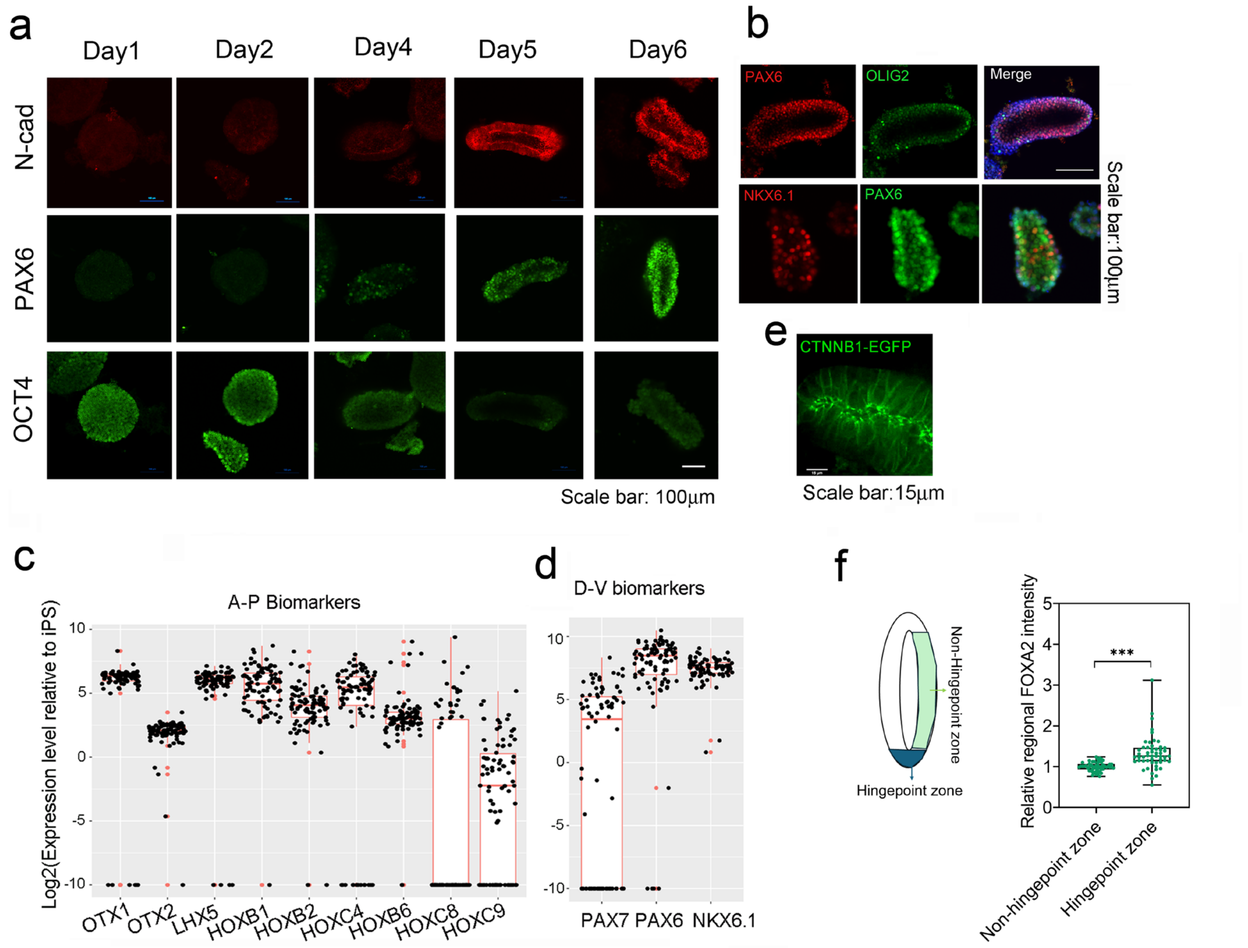

Fig. S5. Human iPS cell-derived neural tube-like organoid characterization.
(**a**) Immunofluorescent staining shows representative temporal expression profile of neural markers N-cad (N-cadherin), PAX6 and pluripotency marker OCT4 in human organoids from day 1 to day 6 after neural induction. Scale bar: 100 μm. (**b**) Immunofluorescent co-staining for dorsal-ventral markers PAX6 (dp3-pMN), and OLIG2 (pMN); NKX6.1 (p2 to floor plate) in organoids. Hoechst is blue. Scale bar: 100 μm. (**c-d**) Box plots represent mRNA expression levels of (**c**) Anterior-Posterior markers or (**d**) Dorsal-Ventral markers in human neural tube-like organoids relative to mRNA levels in iPS cells. (**e**) Membrane distribution of CTNNB1-EGFP throughout the apical-basal axis suggesting pseudostratified epithelium of the human neural tube-like organoids. Scale bar: 15 μm. (**e**) Quantification of FOXA2 intensity in non-hingepoint

region and Hingepoint region in organoids, one-way ANOVA; ***: $p < 0.0001$; n = 53 from four independent experiments for each group.

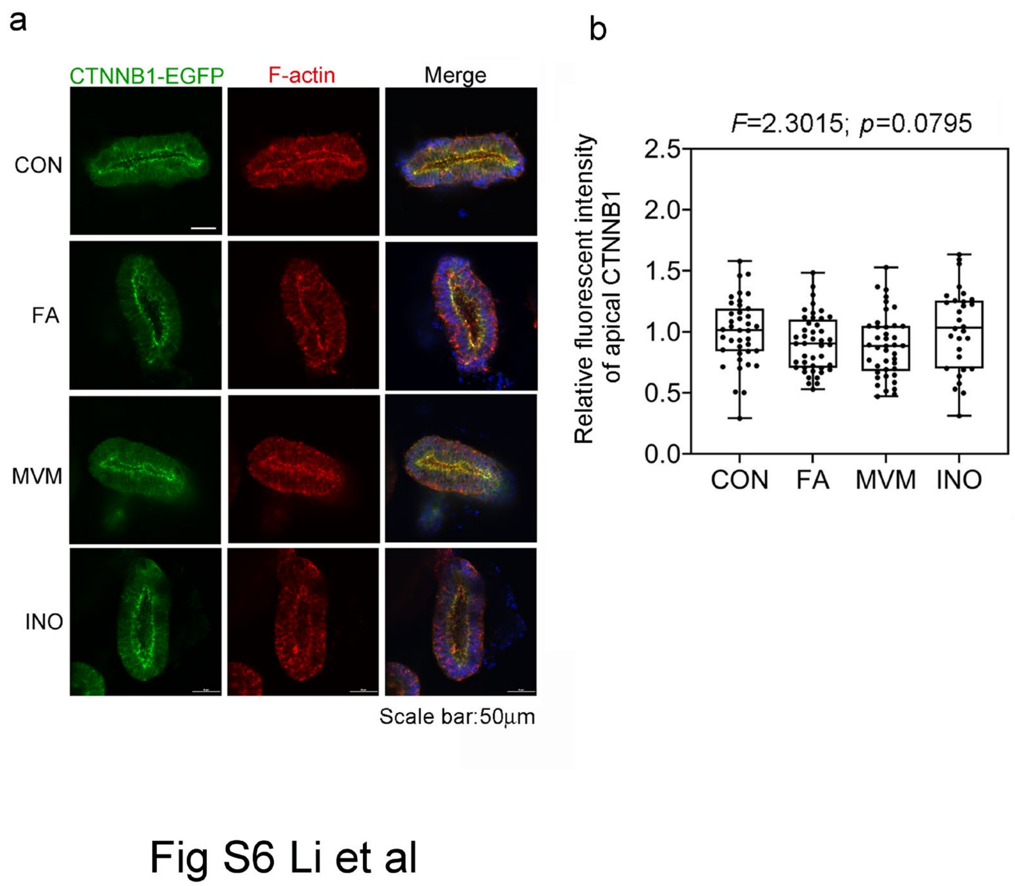

Fig. S6. **Nutrition supplementation does not impact apical adherens junctions in human neural tube-like organoids.**

(**a-b**) Immunofluorescent staining and quantification indicates multivitamins/minerals (MVM), folic acid (FA), or inositol (INO) supplementation does not change the fluorescent intensity of apical CTNNB1. (CON: $n = 39$; FA: $n = 44$; MVM: $n = 41$; INO: $n = 30$ from four independent experiments). Scale bar: 50 μm. Box plots and whisker with all points were shown. One-way ANOVA followed by Post Hoc Tukey HSD.

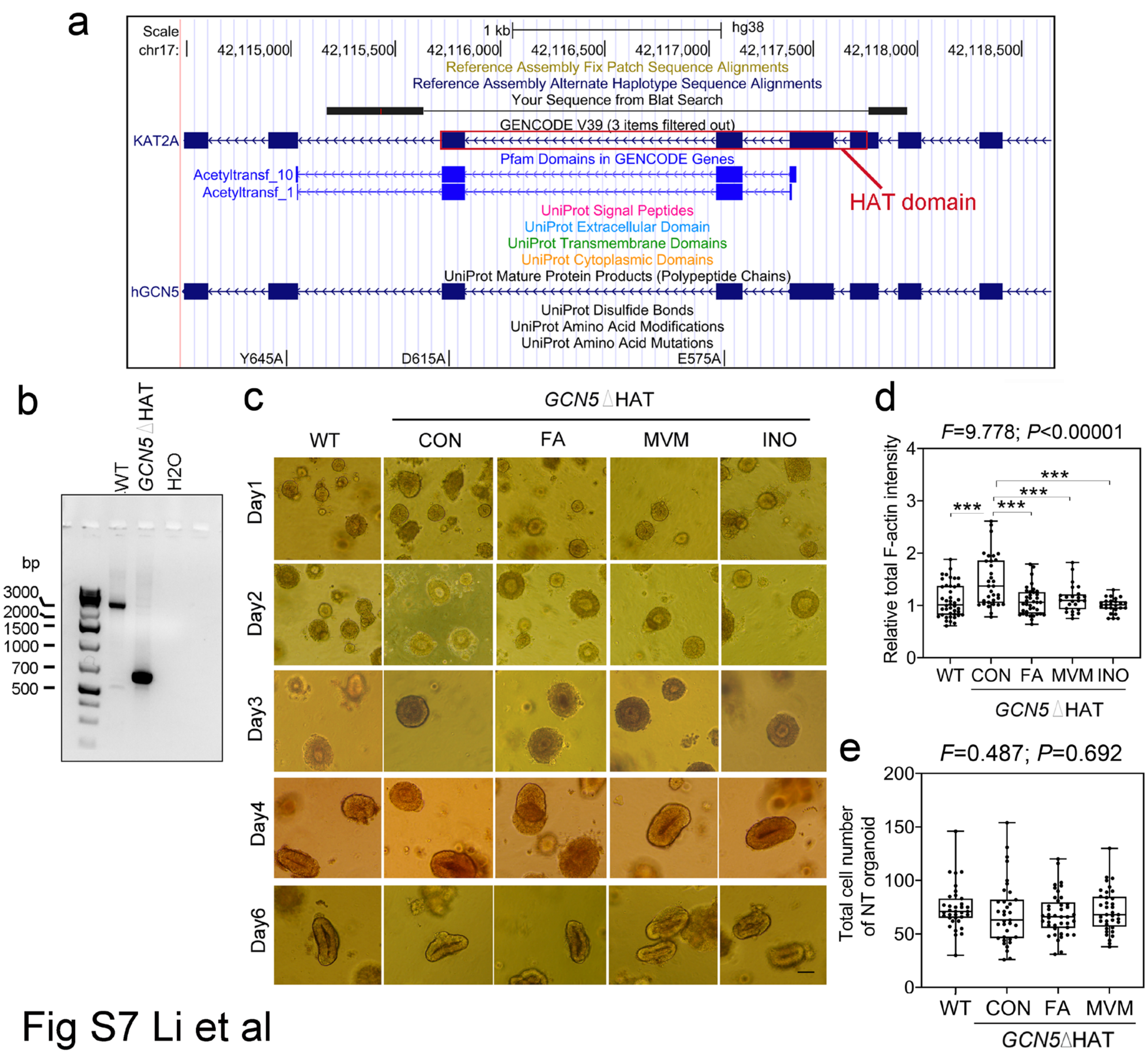

Fig. S7. Creation of *GCN5*△HAT mutant iPSC line for generation of organoids
(**a**) Genome browser view indicates deletion of the HAT domain of human GCN5 protein in the iPSC line. (**b**) The gel image shows the PCR bands following genotyping of WT and *GCN5*△HAT mutant line. (**c**) Bright field images are representative of successful induction of neural tube-like organoids from the *GCN5*△HAT mutant line from induction day1 to day6. (**d**) Quantification of total F-actin intensity in organoids. ***: $p < 0.0001$, One-way ANOVA followed by Post Hoc Tukey HSD. WT: $n = 42$; CON: $n = 33$; FA: $n = 37$; MVM: $n = 24$; INO: $n = 25$ from six independent assays. WT: wild type; CON: control media; FA: Folic acid; MVM: multivitamins/minerals, INO: Inositol. (**e**) Quantification of total cell number (marked by H2B-EGFP) within each wildtype (WT) or *GCN5*△HAT mutant organoid grown in control or nutrient supplemented media (WT: $n = 35$; CON: $n = 35$; FA: $n = 41$; MVM: $n = 36$ from five independent assays) in Figure 4h. Box plots and whisker with all points were shown. One-way ANOVA followed by Post Hoc Tukey HSD. Scale bar in **c**: 100 μm.

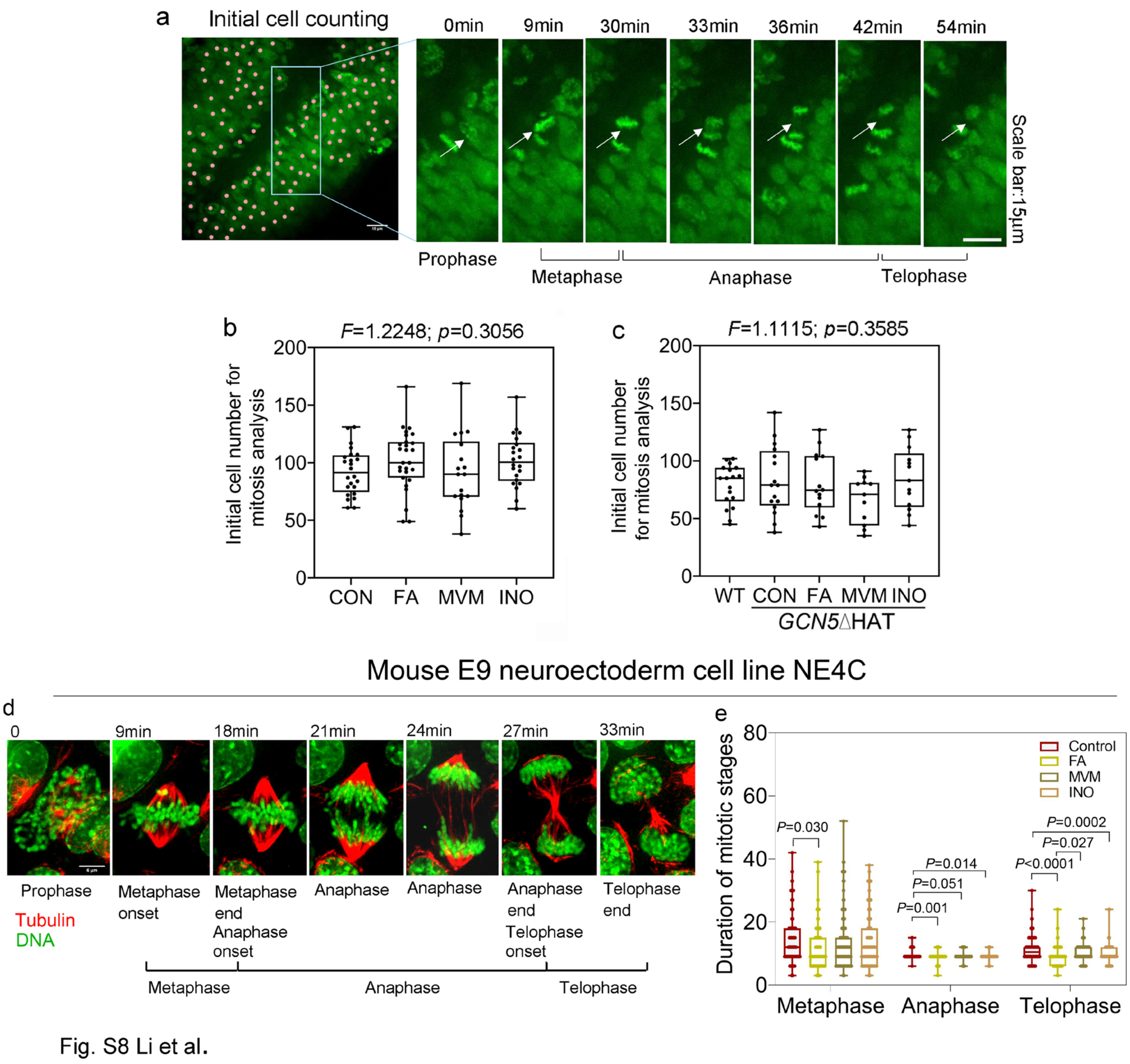

Fig. S8: **MVM supplementation can partially avoid FA alone-elicited abnormal cell mitosis.** (**a**) Representative still images of time-lapse movies show the method of initial cell counting (left panel, dots indicate each cell) and analysis of duration of mitotic stages (right panel) in the organoids. Arrows in right panels point out the representative stage of cell division, which is indicated below the panels. (**b-c**) Quantification of the number of initial cells, used for normalization of the number of mitoses in wildtype organoids (**b**; CON, control media: $n = 24$; FA: $n = 27$; MVM: $n = 19$; INO: $n = 22$ from six independent assays), and in the *GCN5*△HAT mutant organoids (**c**; WT in control media: $n = 18$; CON, control media: $n = 16$; FA: $n = 10$; MVM: $n = 10$; INO: $n = 13$ from four independent assays). One-way ANOVA followed by Post Hoc Tukey HSD. (d) Representative still images of live imaging show the timing of mitosis in NE-4C cells visualized with SPY555-Tubulin (Red) and SPY505-DNA (green). (e)

Quantification of durations of mitotic stages in NE-4C cells supplemented with CON: n = 130; FA: *n* = 127; MVM: *n* = 132; INO: *n* = 97 from three independent assays. Box plots and whisker with all points were shown. One-way ANOVA followed by post-hoc Tukey HSD were used in all quantification data.

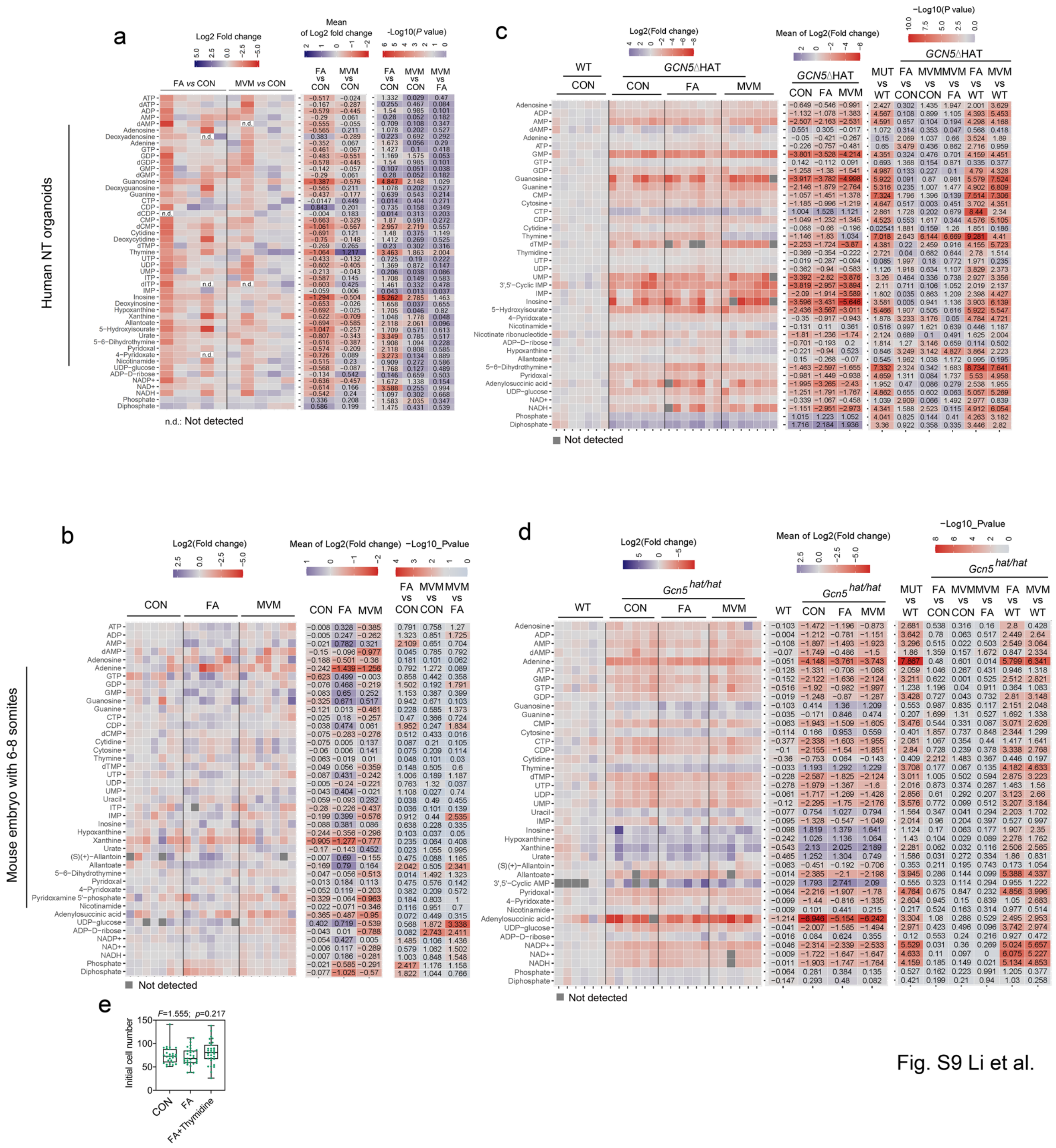

Fig. S9: Nucleotide metabolites of human neural tube organoids or mouse embryos with FA alone or MVM supplementation. (a) The heatmap shows a full list of relative concentrations of nucleotide metabolites in human organoids with FA alone or MVM supplementation versus

control media in Figure 6d (*n* = 5 independent samples) or (**c**) in *GCN5*△HAT mutant organoids supplemented with control medium (CON); folic acid (FA) or multivitamins/minerals (MVM) relative to wildtype (WT) (*n* = 7 independent samples). (**b**) The heatmaps show intracellular concentrations of nucleotide metabolites in whole body of C57BL/6J embryos at 6-8 somites stage supplemented with FA or MVM relative to Control (CON), or (**d**) in $Gcn5^{hat/hat}$ mutant embryos with 6-8 somites stage supplemented with CON; FA or MVM (*n* = 7 embryos for each treatment) relative to wildtype (WT). (**a-d**) Left panel: individual Log2 (fold change) relative to CON (**a** and **c**) or WT (**b** and **d**); Middle panel: Mean of Log2 (fold change); Right panel: -log10 *P* value (Student's *t*-test). (**e**) Initial cell number for the organoid experiments in Figure 6h with FA or FA+Thymidine supplementation. CON: *n* = 51; FA: *n* = 47; FA+Thymidine: *n* = 46 from six independent experiments). Box plots and whisker with all points were shown. One-way ANOVA followed by Post Hoc Tukey HSD. n.d. or gray bar = not detected.

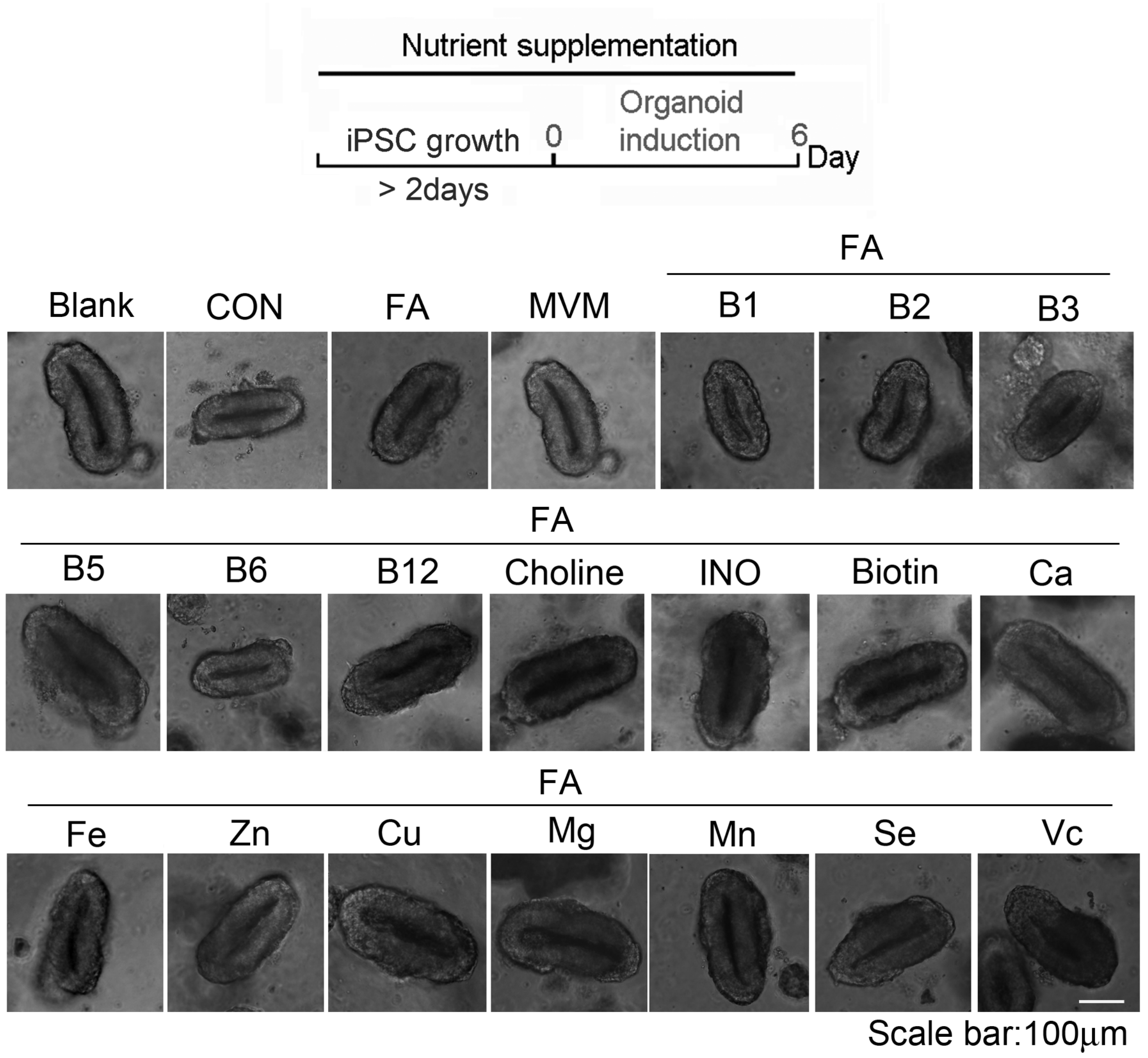

Fig. S10: Bright field images of organoids supplemented with FA and single nutrients

Top panel: schematic of timeline of nutrient supplementation in organoids; other panels: representative images to show appearance of organoids supplemented with FA plus single nutrient. Scale bar: 100 µm. Blank: No supplement; CON: control medium; FA: Folic caid; MVM: multivitamins/minerals.

Video1: Live imaging of a neural tube-like organoid growing from induction day5 to day6. Red: SPY-555-actin; green: H2B-EGFP. Interval: 10 min. Duration 12 hrs.

Video 2: Representative live imaging for interkinetic nuclear migration in a human neural tube-like organoid using the H2B-EGFP cell line. Interval: 3 min. Duration 66 min.

Video 3: Representative live imaging for the number of mitoses in human neural tube-like organoid from H2B-EGFP cell line grown in control media. Interval: 3 min. Duration 3 hrs.

Video 4: Representative live imaging for the number of mitoses in human neural tube-like organoid from H2B-EGFP cell line supplemented with folic acid alone. Interval: 3 min. Duration 3 hrs.

Video 5: Representative live imaging for the number of mitoses in human neural tube-like organoid from H2B-EGFP cell line supplemented with multivitamins/minerals. Interval: 3 min. Duration 3hrs.

Video 6: Representative live imaging for the number of mitoses in human neural tube-like organoid from H2B-EGFP cell line supplemented with inositol alone. Interval: 3 min. Duration 3 hrs.

Video 7: Representative live imaging for the number of mitoses in wildtype human neural tube-like organoid from H2B-EGFP cell line. Interval: 3 min. Duration: 3 hrs.

Video 8: Representative live imaging for the number of mitoses in *GCN5*△HAT mutant human neural tube-like organoid from H2B-EGFP cell line in control media. Interval: 3 min. Duration: 3 hrs.

Video 9: Representative live imaging for the number of mitoses in *GCN5*△HAT mutant human neural tube-like organoid from H2B-EGFP cell line supplemented with folic acid alone. Interval: 3 min. Duration: 3 hrs.

Video 10: Representative live imaging for the number of mitoses in *GCN5*△HAT mutant human neural tube-like organoid from H2B-EGFP cell line supplemented with multivitamins/minerals. Interval: 3 min. Duration: 3 hrs.

Video 11: Representative live imaging for the number of mitoses in *GCN5*△HAT mutant human neural tube organoid supplemented with inositol alone in H2B-EGFP cell line. Interval: 3 min. Duration: 3 hrs.
